# The Protective Effect of *Trichilia catigua* A. Juss. on DEHP-Induced Reproductive System Damage in Male Mice

**DOI:** 10.3389/fphar.2022.832789

**Published:** 2022-02-03

**Authors:** Xinyue Chang, Mingran Dong, Xiao Mi, Meigeng Hu, Juan Lu, Xi Chen

**Affiliations:** Institute of Medicinal Plant Development, Chinese Academy of Medical Sciences, Peking Union Medical College, Beijing, China

**Keywords:** *Trichilia catigua*, DEHP, phenolic, reproductive system, oxidative damage

## Abstract

The present study aimed to explore the protective effect and molecular mechanisms of *Trichilia catigua* A. Juss. extract (TCE) against di (2-ethylhexyl) phthalate (DEHP)-induced damage to the reproductive system of mice. Acute toxicity tests revealed that the maximum tolerated dose (MTD) in mice was up to 2.7 g kg^−1^. After induction with DEHP, TCE (L-TCE, M-TCE, H-TCE) was orally administered to mice for 28 days. Differences in indicators among groups showed that TCE significantly improved the anogenital distance and the organ indexes of the epididymides and testes. It also significantly reduced varicocele and interstitial cell lesions compared to the model group. H-TCE reduced the sperm abnormality rate, increased the levels of sex hormones, Na^+^K^+^ and Mg^2+^, Ca^2+^-ATPase enzyme activity, antioxidant enzyme vitality, coupled with a significant decrease in LH and MDA contents. The levels of testicular marker enzymes ACP and LDH were significantly augmented by both M-TCE and H-TCE. Further studies claimed that DEHP induction reduced the mRNA expression levels of *Nrf2, SOD2, SOD3, CDC25C CDK1*, *CYP11A1, 3β-HSD, 5ɑ-R, AR, SF1,* and *CYP17A1,* increased the level of *Keap1*, while TCE reversed the expression levels of these genes. Meanwhile, IHC results demonstrated a significant change in the expression activity of the relevant proteins compared to the control group. The results suggest that M-TCE and H-TCE enabled the recovery of DEHP-induced reproductive system damage in male mice by improving testicular histopathology, repairing testicular function, and reducing oxidative stress damage. The oxidation-related Keap1-Nrf2 pathway, SODs enzyme, the cell cycle control-related CDC25C-CDK1 pathway, and the steroidogenic-related pathway may contribute to this protective effects of TCE.

**GRAPHICAL ABSTRACT:**
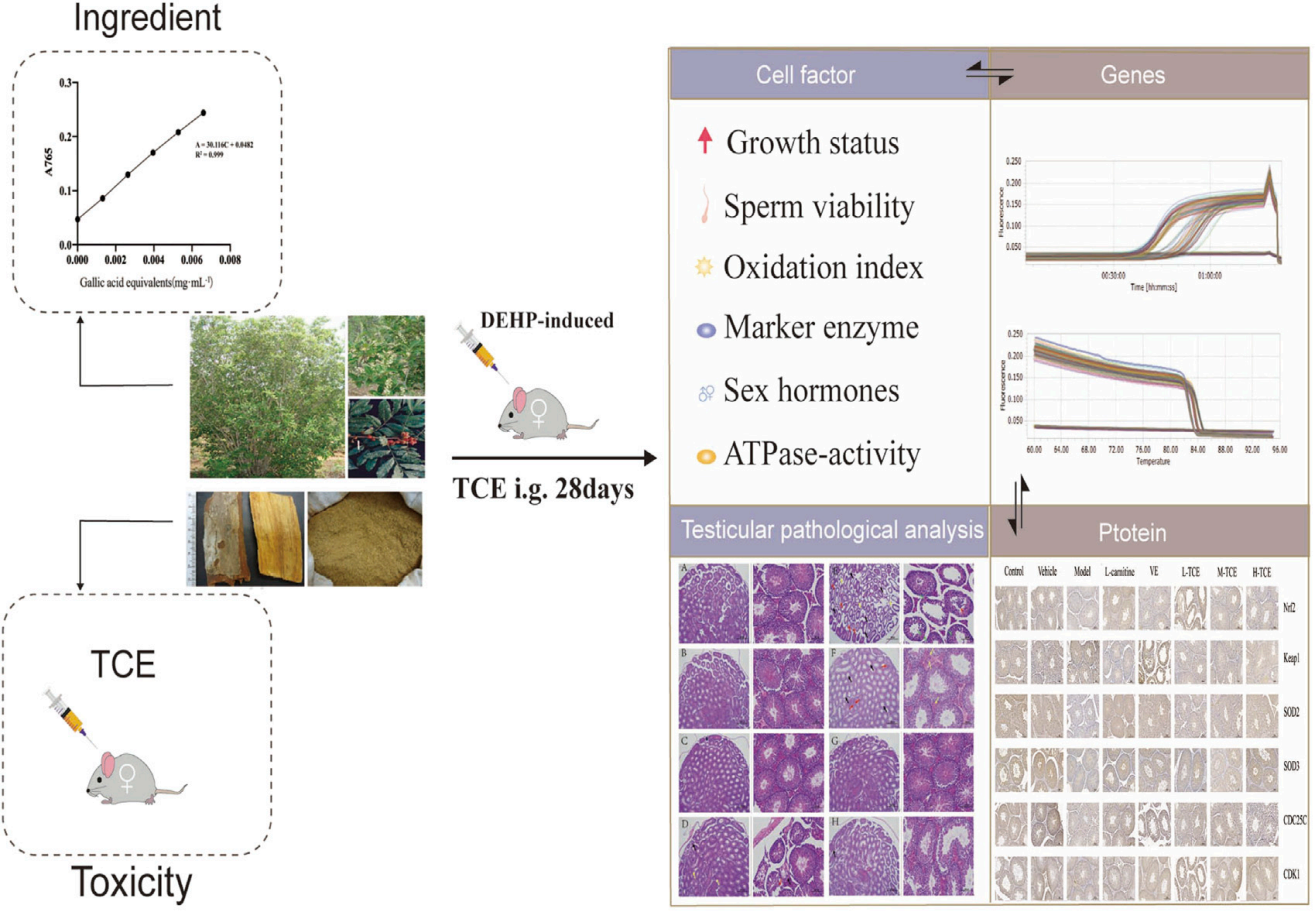


## Introduction

Several predisposing factors that are responsible for the decline in sperm quality and count have been reported in the past 50 years (Damstra et al., 2002; Merzenich et al., 2010). Primarily, environmental triggers and lifestyle, including endocrine-disrupting chemicals, can significantly impact male reproductive health (Street et al., 2018). One of the most widely used plasticizers, di (2-ethylhexyl) phthalate (DEHP), can enter the body through drinking, eating, skin contact (cosmetics), and breathing. The estrogen-like effects of DEHP affect the normal secretion of hormones in living organisms, causing varying degrees of harm to human health (Farombi et al., 2007; Muczynski et al., 2012). Studies have reported that DEHP interferes with the reproductive endocrine system, reducing sperm motility, decreasing sperm count, causing atrophy and morphological abnormalities in parenchymal organs such as the testis, and is thus highly toxic to the male reproductive system (Saunders et al., 1997; Lyche et al., 2009; Abd-Ellah et al., 2016). The pathophysiological mechanism behind phthalate-induced atrophy in several tissues may be attributed to oxidative stress (Farombi et al., 2007; Muczynski et al., 2012). These are involved in deregulation in the balance of antioxidant defense in tissues and induction of peroxidative damage (Andrade et al., 2006). Several studies have documented that oxidative damage induced by phthalates can lead to DNA damage and impaired mitochondrial function, reducing the activity of genes associated with high pass synthesis, impeding organ development and testicular sperm production (Yeung et al., 2011; Barakat et al., 2017; Barakat et al., 2019).

*Trichilia catigua* A. Juss. is a small shrubby tree prevalent in South America. It is commonly used locally in Brazil as an aphrodisiac tonic. Preparations of this herb also find application in folk medicine to alleviate fatigue, stress, and improve memory. Recent research has confirmed several pharmacological activities, including antioxidant, antinociceptive, anti-inflammatory, and neuroprotective effects of *Trichilia catigua* (Silva, 2004; Longhini et al., 2017). Alcoholic extract of the *Trichilia catigua* bark, traditionally known as “Catuama” (Pizzolatti et al., 2002), exhibits significant effects in stimulating erection time in the rabbit cavernosa and is generally exploited as a stimulant of the reproductive system and as an anti-fatigue drug (Antunes et al., 2001; Silva, 2004). Available studies indicate the safety of *Trichilia catigua* for use in healthy human volunteers, with no known side effects or adverse effects (Oliveira et al., 2005). The presence of several secondary metabolites, including omega-phenyl alkanes, omega-phenyl alkanoic acids, omega-phenyl-gamma-lactones, alkyl-gamma-lactones, alkenyl-gamma-lactones, fatty acids, b-sitosterol, stigmasterol, campesterol, epicatechin, cinchonains (Ia, Ib, IIa, IIb), catiguanins A and B, procyanidins B2 and C1, tannins, and a mixture of flavalignans, has been reported by phytochemical studies on *Trichilia catigua* (Tang et al., 2007).

*Trichilia catigua* finds widespread application in folk medicine to treat free radical-related diseases. Modern pharmacological research has attempted to determine its antioxidant activity in a neurological context. Nonetheless, there is little information in the literature about *Trichilia catigua’s* antioxidant properties on the reproductive system. We, therefore, employed DEHP to construct a model of reproductive system oxidative stress damage to investigate the reproductive system antioxidant damage activity of ethanol extracts from the stem and bark of *Trichilia catigua*.

## Materials and Methods

### Equipment and Reagents

The equipment used for this study was as follows: AB265-S Analytical Balance (METTLER TOLEDO, Switzerland), BCD-206TS Haier Refrigerator (Qingdao Haier Co., Ltd.), DZKW-4 Electronic Thermostatic Water Bath [Linmao Technology (Beijing) Co., Ltd.], FJ-200 High-Speed Dispersion Homogenizer (Shanghai Specimen Model Factory), NanoDrop 2000 Spectrophotometers (Thermo Fisher Scientific Inc., MA, United States), LightCycler^®^ 480 Real-Time System (Roche, United States), Biological microscopes (Nikon, eclipse Ci., Japan), MDF-U53V ultra-low temperature refrigerator (SANYO, Japan).

The following reagents were employed in the present study: *Trichilia catigua* A. Juss. was provided by FITOTERAPIA BIOTECH LTDA, collected in Bahia, Northeast Brazil, was identified by Li Haitao, Yunnan Branch, Institute of Medicinal Plants, Chinese Academy of Medical Sciences. Folin-Phenol (F8060, Solarbio). Gallic acid (151017) was purchased from Shanghai Winherb Medical Technology Co., Ltd. (Shanghai, China). The purity of standards chemicals ≥98%. DEHP (Alfa Aesar, A10415), Superoxide dismutase (SOD) Activity Assay (A001), Malondialdehyde (MDA) kit (A003), Catalase (CAT) assay kit (A007), Reduced glutathione (GSH) assay kit (A006), Glutathion reductases (GR) assay kit (A062), Glutathione peroxidase (GSH-Px) kit (A005), Succinate dehydrogenase (SDH) kit (A022), Acid phosphatase (ACP) assay kit (A060), Lactate dehydrogenase (LDH) kit (A020), Alkaline phosphatase (ALP/AKP) assay kit (A059) were all purchased from Nanjing Jiancheng Technology Co., Ltd. (Nanjing, China). Mlbio Mouse follicle-stimulating hormone (FSH) ELISA Kit (ml001910), Mlbio Mouse estradiol (E2) ELISA Kit (ml001962), Mlbio Mouse testosterone (T) ELISA Kit (ml001948), and Mlbio Mouse luteinizing hormone (LH) ELISA Kit (ml001948) were bought from Shanghai enzyme-linked Biotechnology Co., Ltd. (Shanghai, China). RNAiso Plus (TaKaRa Bio, Lot No. 9108–9109); Prime Script RT Master Mix (TaKaRa Bio, Lot No. DRR036A); TB Green Premix Ex Taq (TaKaRa Bio, Lot No. RR820WR).

### Animal

Male ICR mice (18–22 g) were obtained from Beijing HFK Bioscience Co., Ltd. (Beijing, China). The research animal ethics committee of the Institute of Medicinal Plant Development affiliated with the Chinese Academy of Medical Sciences (Beijing, China) approved all animal-related protocols that were performed according to the National Institutes of Health guide. Animals were kept in a semi-barrier system at room temperature and humidity with a 12–12 h/light-dark photoperiod. All mice were acclimatized to the environment for 1 week before experimentation. The experimental protocol was approved by the institutional ethics committee (approval no. SLXD-20201225042 and SLXD-20210507012).

### Plant Material and Extract Preparation

As per previous studies, the bark of *Trichilia catigua* was roughly extracted with water (Bernardo et al., 2018; Panizzon et al., 2019), 50% (Barbosa et al., 2004), 70% (Kamdem et al., 2012), and 95% (Pizzolatti et al., 2002) ethanol solutions to obtain four crude extracts, respectively. Subsequent extraction of these alcoholic extracts with petroleum ether, ethyl acetate, and n-butanol retrieved the corresponding nine organic phase components for a total of 13 extracts. The cell viability and the degree of oxidation of TM_4_ cells damaged by H_2_O_2_ were compared. The results showed that the ethyl acetate fraction of the 50% alcoholic extract provided adequate protection against oxidative damage to germ cells. Therefore, we selected this component for our study.

The preparation process was as follows: 1,000 ml of 50% ethanol solution was added to 100 g of *Trichilia catigua* bark powder with the ratio of material to liquid = 1:10, thoroughly stirred, followed by cold macerate twice, each time for 24 h, and filtration. The two extracts were then combined, concentrated, and dried to obtain the crude extract. The crude extract was shaken with 300 ml of distilled water to disperse it evenly in the aqueous solution, extracted with petroleum ether and ethyl acetate in turn, collected to obtain the corresponding ethyl acetate fraction, and after that spin-dried again to obtain TCE. After repeated extraction, the calculated yield of TCE powder was about 4.21%

### Estimation of Total Phenolic Content

#### Preparation of Sample Solution

To prepare the control stock solution, 2.50 mg of gallic acid was weighed in a 25 ml volumetric flask, and the stock solution was fixed with distilled water to the scale.

Similarly, the TCE stock solution was prepared by weighing 25.00 mg of TCE in a 25 ml volumetric flask, and the stock solution was fixed with distilled water to the scale.

#### Establishment of the Gallic Acid Standard Curve

The total phenolic content was determined by employing the methods given in the literature (Slinkard and Singleton, 1977) with some modification. Varied amount of the control stock solution (0, 0.25, 0.5, 0.75, 1.0, 1.25 ml) was pipetted into a 25 ml volumetric flask and 1.5 ml of forintanol reagent was added. After 8 min, 1.5 ml of 10% Na_2_CO_3_ solution was added to the mixture, and the reaction was carried out at 25°C for 1 h. The absorbance was recorded at 765 nm. The linear regression equation was obtained with A as absorbance and C as gallic acid concentration.

#### Estimation of Total Phenolic Content

Following the method under 2.4.2, 1.5 ml from the sample stock solution was taken in a 25 ml volumetric flask and the absorbance was determined at 765 nm. The absorbance was repeated three times. With the help of the standard curve, the absorbance values were then employed to calculate the total polyphenol content of the sample.

### Acute Toxicity Test in Mice

Corn oil was used as a co-solvent to configure a 900 mg kg^−1^ TCE solution and diluted step by step to prepare a diluted sample solution of the appropriate concentration. Thirty-six male ICR mice (18–22 g) were randomly and evenly distributed into six groups (*n* = 6), including control [distilled water, intragastric administration (i.g.)] and different dosed groups (178 mg kg^−1^, 267 mg kg^−1^, 400 mg kg^−1^, 600 mg kg^−1^, 900 mg kg^−1^ TCE, i.g.). Animals were treated for 7 days.

The mice were scrutinized for appearance, behavior, secretions, excretions, and mortality; LD50 was determined by mortality. If the LD50 value cannot be measured, dosing at 900 mg kg^−1^ was repeated, and the MTD value was determined.

### *In vivo* Experiments

#### Animal Grouping and Dosing

The solution was prepared as directed in 2.5. Ninety-six male ICR mice (18–22 g) were randomly and evenly classified into eight groups (*n* = 12), including Control (distilled water), Vehicle (corn oil), model (2.5 mg g^−1^ DEHP daily for 7 days, i.g.), L-carnitine (DEHP for 7 days then 500 mg kg^−1^ L-carnitine, i.g.), VE (DEHP for 7 days then 100 mg kg^−1^ VE, i.g.), L-TCE, M-TCE, and H-TCE (DEHP for 7 days then 300, 600, 900 mg kg^−1^ TCE, i.g.). Treatment was continued for 28 days. The DEHP-induced method has been described in the Supplementary Material.

#### Sample Collection

The animals fasted overnight at the end of the experiment. On the 28th day, the animals were weighed and the anogenital distance (the distance between the penile and anal openings) was measured. Blood samples were centrifuged at 3,500 rpm for 10 min, serum was isolated and stored at −80°C until use. The testes and epididymides samples were removed intact bilaterally, the surrounding fat and connective tissue were stripped and then weighed, and the organ indexes {Organ index = [organ mass (g)/body mass (g)] * 100%} was calculated. Excising the left testis of each mouse, it was immersed in 4% paraformaldehyde for HE staining and IHC, while the right testis was placed in liquid nitrogen to prepare tissue homogenates for the determination of various parameters.

#### Testicular Pathological Analysis

Small pieces were removed from the caudal extremity of the testes and fixed in 10% neutral buffered formalin. Proper fixation was followed by dehydration of the specimens in graded ethanol, clearing in xylene, embedding in paraplast, and sectioning at 3–5 µm thickness. The sections were stained using the HE staining method to demonstrate the histological structure of testes in both control and DEHP-induced animals. A Leitz Dialux 20 microscope was adopted to analyze the stained sections. Images were taken using a Canon digital camera (Canon PowerShot A95). Slides were then evaluated for cellular changes in the spermatogenic epithelium.

#### Determination of Sperm Count and Sperm Abnormality Rate

To estimate the sperm count and abnormality rate, the bilateral epididymides was placed in 3 ml of saline pre-warmed to 37°C, cut up, and placed at 37°C for 1 min before making a sperm suspension.

Determination of sperm count: A drop of sperm suspension was poured into the counting cell (0.1 mm^3^) of the hematocrit plate, the number of sperm in five squares of the counting cell (n) was counted. The total number of sperm in 1 ml (1,000 mm^3^) of sperm suspension is 50,000n.

Determination of sperm abnormality rate: The sperm suspension was centrifuged at 1,000 r/min for 5 min, the supernatant was discarded, and the precipitate was mixed with a small amount of the remaining liquid. A suspension drop was placed on a slide and pushed away, dried and fixed in methanol for 5 min, and stained with 2% eosin aqueous solution for 1 h. The morphology of 1,000 sperm was examined to compute the abnormality rate (%).

#### Determination of Sex Hormones

The concentrations of T, LH, FSH, and E2 in serum were determined as per the instructions of the assay kit. The concentration of the standard served as the horizontal coordinate, whereas the corresponding OD value represented the vertical coordinate to plot a linear regression curve for each hormone. The serum samples were diluted to a certain concentration, and the concentrations were calculated using the standard curve.

#### Determination of AKP, ACP, and LDH

The assay kit instructions were followed to measure the testicular marker enzymes AKP, ACP, and LDH in testicular tissue homogenates.

#### Determination of ATPase

The assay kit instructions were followed to measure ATPase activity in testicular tissue homogenates.

#### Determination of SOD, MDA, GSH-Px, GSH, GR, and CAT

SOD, MDA, and GSH-Px in testicular tissue homogenates were assessed according to the kit instructions.

### qRT-PCR Detection of Gene Expression

Testicular tissue samples were collected from the mice as described in 2.6.2, followed by total RNA extraction according to the RNAiso Plus Kit protocol. The total RNA was reverse transcribed into cDNA to estimate its concentration and purity. The synthesized cDNA was subsequently subjected to qRT-PCR analysis. The LightCycler^®^ 480 Real-Time System was adopted to conduct qPCR and measure the relative expression of the mRNA. The primer sequences for mice *Gapdh* (the housekeeping gene), *Nrf2, HO-1, Keap1, INOS, SOD1, SOD2, SOD3, CAT, PGC1α* (the oxidation-related gene), and *CDC25A, CDC25C, CDK1, Wee1* (the cell cycle control-related gene) and *star, P450scc, CYP11A1, CYP17A1, 3β-HSD, 5ɑ-R, AR, SP1, SF1* (the steroidogenic-related gene) are detailed in Table 1.

**TABLE 1:** Primers for real-time PCR analyses.

Gene	Species	Forward (5′-3′)	Reverse (3′-5′)
*GAPDH*	Mouse	AGG​TCG​GTG​TGA​ACG​GAT​TTG	TGT​AGA​CCA​TGT​AGT​TGA​GGT​CA
*Nrf2*	Mouse	TAG​ATG​ACC​ATG​AGT​CGC​TTG​C	GCC​AAA​CTT​GCT​CCA​TGT​CC
*H O -1*	Mouse	GAT​AGA​GCG​CAA​CAA​GCA​GAA	CAG​TGA​GGC​CCA​TAC​CAG​AAG
*Keap1*	Mouse	TCG​AAG​GCA​TCC​ACC​CTA​AG	CTC​GAA​CCA​CGC​TGT​CAA​TCT
*INOS*	Mouse	GTT​CTC​AGC​CCA​ACA​ATA​CAA​GA	GTG​GAC​GGG​TCG​ATG​TCA​C
*SOD1*	Mouse	AAC​CAG​TTG​TGT​TGT​CAG​GAC	CCA​CCA​TGT​TTC​TTA​GAG​TGA​GG
*SOD2*	Mouse	CAG​ACC​TGC​CTT​ACG​ACT​ATG​G	CTC​GGT​GGC​GTT​GAG​ATT​GTT
*SOD3*	Mouse	CCT​TCT​TGT​TCT​ACG​GCT​TGC	TCG​CCT​ATC​TTC​TCA​ACC​AGG
*CAT*	Mouse	TGG​CAC​ACT​TTG​ACA​GAG​AGC	CCT​TTG​CCT​TGG​AGT​ATC​TGG
*PGC1α*	Mouse	TAT​GGA​GTG​ACA​TAG​AGT​GTG​CT	GTC​GCT​ACA​CCA​CTT​CAA​TCC
*CDC25A*	Mouse	TTT​TGG​ACA​GTG​ACC​CAA​GAG​A	TCT​TTA​ATG​AGA​CCG​GCA​AAC​TT
*CDC25C*	Mouse	GTT​TCA​GCA​CCC​AGT​TTT​AGG​T	AGA​ATG​CTT​AGG​TTT​GCC​GAG
*CDK1*	Mouse	AAA​TCC​TCC​AGG​GAA​TTG​TGT​TT	CAG​CCA​GTT​TGA​TTG​TTC​CTT​TG
*Wee1*	Mouse	TTC​CGG​CTC​TGT​TAA​ACT​CCG	CGA​CAC​CGT​CCT​GAG​GAA​TG
*star*	Mouse	AAC​GGG​GAC​GAA​GTG​CTA​AG	CCG​TGT​CTT​TTC​CAA​TCC​TCT​G
*P450scc*	Mouse	AGG​TCC​TTC​AAT​GAG​ATC​CCT​T	TCC​CTG​TAA​ATG​GGG​CCA​TAC
*CYP11A1*	Mouse	AGG​TCC​TTC​AAT​GAG​ATC​CCT​T	TCC​CTG​TAA​ATG​GGG​CCA​TAC
*CYP17A1*	Mouse	GTC​GCC​TTT​GCG​GAT​AGT​AGT	TGA​GTT​GGC​TTC​CTG​ACA​TAT​CA
*3β-HSD*	Mouse	GGC​AAA​TTC​TCC​ATA​GCC​AA	GCT​TCC​TCC​CAG​TTG​ACA​AG
*5ɑ-R*	Mouse	GAG​TTG​GAT​GAG​TTG​CGC​CTA	GGA​CCA​CTG​CGA​GGA​GTA​G
*AR*	Mouse	CAG​GAG​GTA​ATC​TCC​GAA​GGC	ACA​GAC​ACT​GCT​TTA​CAC​AAC​TC
*SP1*	Mouse	AGG​GTC​CGA​GTC​AGT​CAG​G	CTC​GCT​GCC​ATT​GGT​ACT​GTT
*SF1*	Mouse	GAG​AGT​TCC​GTA​CCC​GCA​AAA	CTC​TGG​GTC​CAA​TTA​GGA​GCC
*Occludin*	Mouse	TGA​AAG​TCC​ACC​TCC​TTA​CAG​A	CCG​GAT​AAA​AAG​AGT​ACG​CTG​G
*Z O -1*	Mouse	GCT​TTA​GCG​AAC​AGA​AGG​AGC	TTC​ATT​TTT​CCG​AGA​CTT​CAC​CA

### IHC Studies

The collected testis samples were paraffin sectioned and cut into 5 m thick sections. After blocking endogenous peroxidase, the tissue sections were incubated overnight at 4°C with the primary antibody. The sections were further incubated separately with the secondary antibody for 50 min at room temperature, developed using DAB chromogenic solution, hematoxylin re-stained cell nuclei, and then dehydrated for analysis. The images were photographed with microscopes and analyzed with Image-pro plus 6.0 (Media Cybernetics, Inc., Rockville, MD, United States). The secondary antibody batch numbers and dilutions are listed in Table 2.

**TABLE 2:** Secondary antibody information and dilutions.

Name	Brand	Specie	Batch No.	Dilution
Anti-KEAP1 antibody	Bioss	Rabbit	bs-3648R	1:400
Anti-Nrf2antibody	Bioss	Rabbit	bs-1074R	1:400
Anti-CDC25C antibody	Bioss	Rabbit	bs-9597R	1:200
Anti-SOD2 antibody	Bioss	Rabbit	bs-20667R	1:300
Anti-SOD3 antibody	Bioss	Rabbit	bs-3895R	1:300
Anti-CDK1 antibody	Bioss	Rabbit	bs-1341R	1:300

### Statistical Analysis

All experiments were independently repeated at least three times. Data were expressed as the mean ± SD. One-way analysis of variance (ANOVA) was adopted to scrutinize the quantitative data. *p* < .05 was considered statistically significant (*p < .05; **p < .01).

## Results

### Estimation of Total Phenolic Content

#### Establishment of the Gallic Acid Standard Curve

**FIGURE 1:**
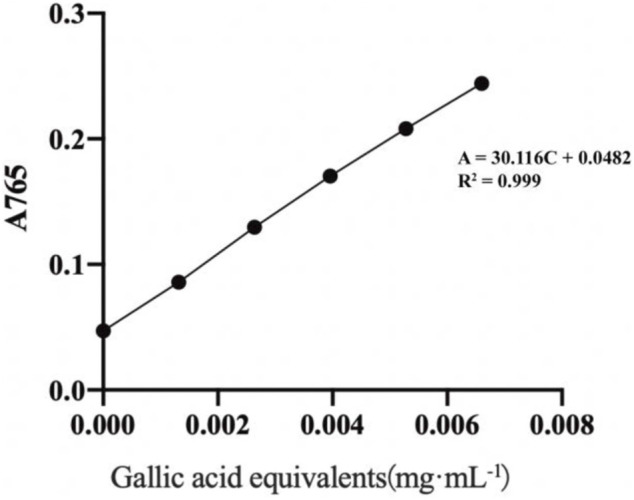
Standard curve of gallic acid.

As illustrated in Figure 1, a linear regression equation was obtained: A = 30.116C + 0.0482, *R* ^2^ = 0.999, with good linearity over the concentration range of (0.001–0.066) mg·mL^−1^ for gallic acid.

#### Estimation of Total Phenolic Content

Applying the absorbance value to the standard curve equation, a total polyphenol content of (41.07 ± 1.63) mg GAE·g^−1^ was obtained.

### Result of Acute Toxicity Test

#### Pre-Experimental Results

After administration, symptoms such as curling, twitching of the limbs, and cramps were observed in mice within 0.75 h of the maximum dose molding, which lasted for a certain period. The mice manifested signs of idleness, quietness, and reduced activity 1 h post-administration. Some mice also exhibited signs of toxicity, such as diarrhea. The earliest time of death was 0.75 h after administration. The symptoms reduced within 2–3 days among the surviving mice, and they returned to normal in about 4–5 days.

The mortality rate of the 50% alcoholic ethyl acetate fraction was less than 50% within 7 days. The volume and concentration of the drug administered made it difficult to estimate the LD50 value, so a positive MTD was determined based on the pre-test results.

#### Results of MTD Determination

Following three repeated doses of 900 mg kg^−1^ TCE within 24 h, mice demonstrated signs of toxicity and two mice died within 6 h after the three doses. The approximate MTD of TCE in normal mice was 2.7 g kg^−1^·d^−1^.

### Results of *in vivo* Efficacy Tests

#### Results of the Anogenital Distance, Epididymides, and Testis Indexes

**FIGURE 2:**
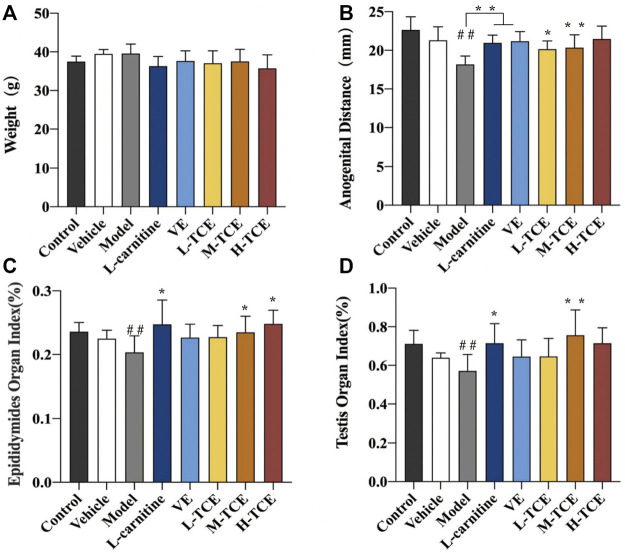
Changes in body weight, anal anogenital distance, epididymides and testicular organ index in different groups **(A)**. Weight; **(B)**. Anogenital distance; **(C)**. Epididymides organ index; **(D)**. Testicular organ index; Means ± SD, *n* = 12; *p < .05, compared with the model group; **p < .01, compared with the model group; ^##^ *p* < .05, compared with the control group; Control: distilled water group; Vehicle: corn oil group; Model: DEHP group; L-carnitine, VE: positive drugs group; L-TCE, M-TCE, H-TCE: low-/middle-/high-dose groups of TCE.

As evident from Figure 2, a significant decrease in the anogenital distance, epididymides, and testis indexes were noted in the model group compared with the control group (*p* < .05). The indexes in the M-TCE groups were significantly elevated relative to the model group (*p* < .05). The H-TCE groups also demonstrated a significant increase in the epididymides index (*p* < .05). Moreover, a significantly higher anogenital distance was observed in the L-TCE groups than in the model group (*p* < .05).

#### Testicular Pathological Analysis

**FIGURE 3:**
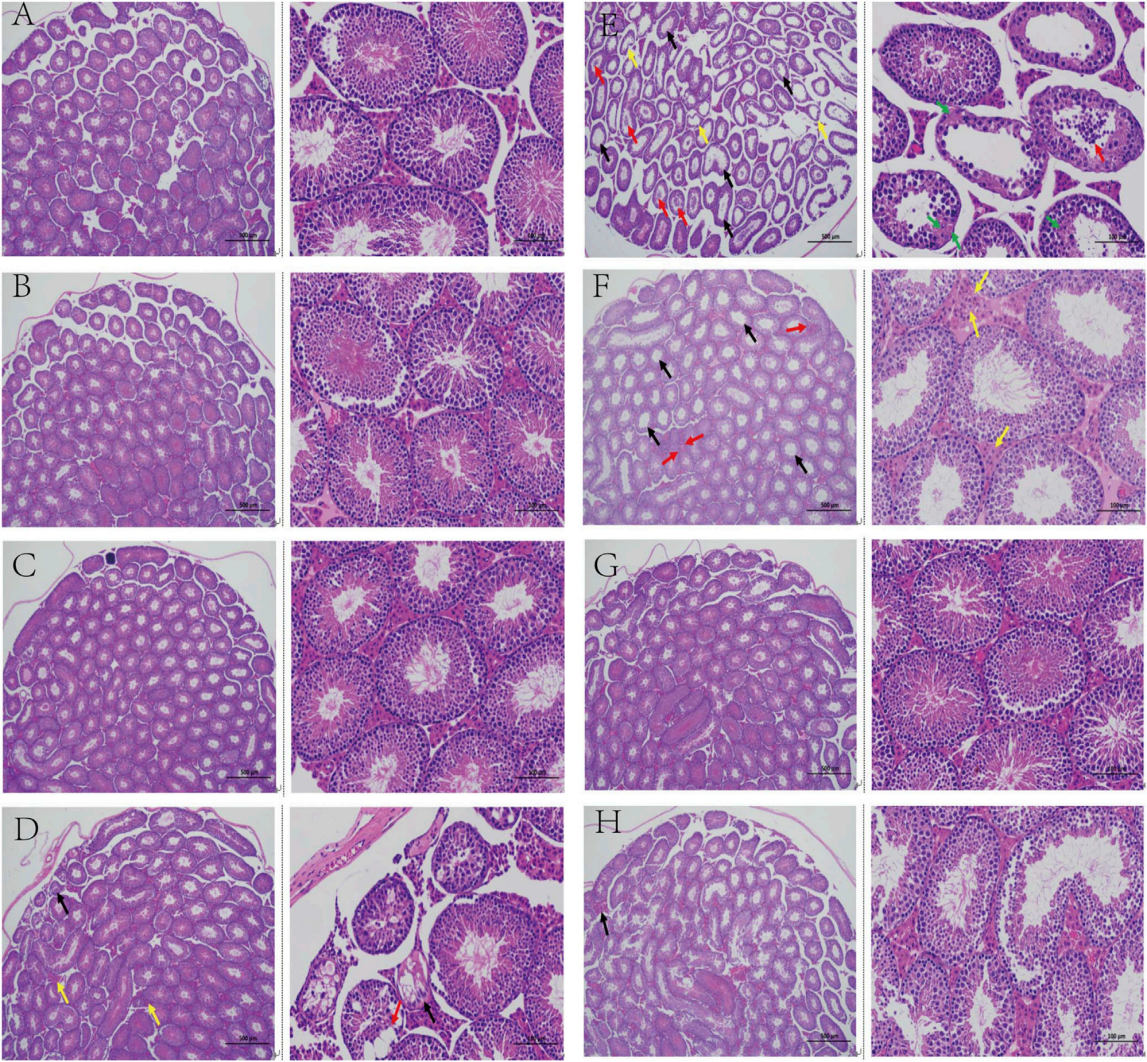
Changes in the testicular pathological analysis in different groups **(A)**. Control group; **(B)**. Vehicle group; **(C)**. L-carnitine group; **(D)**. VE group; **(E)**. Model group; **(F)**. L-TCE group; **(G)**. M-TCE groups; **(H)**. H-TCE groups(x40,x200).

The tubule epithelium is made up of spermatogenic and supporting cells, as shown in Figure 3. The cells in the control group had normal morphology, were neatly arranged and dense, and contained a lot of sperm in the seminiferous tubules. Compared to the control group, the testicular tissue structure of mice in the model group revealed a small number of atrophied varicoceles, with massive loss of germ cells in the tubules (black arrows), shedding of germ cells in the lumen (red arrows), atrophy of the varicocele and loss of germ cells (yellow arrows), necrosis of germ cells in some of the lumen, fragmentation and lysis of the nucleus, and increased eosinophilia of the cytoplasm (green arrows). Limited improvement in testicular pathology with relief of varicocele dilation, atrophy, and interstitial cell hyperplasia was recorded in the L-TCE, M-TCE, and H-TCE groups, with the best improvement reported by the M-TCE group.

#### Result of Total Sperm Count and Sperm Abnormality Rate

**FIGURE 4:**
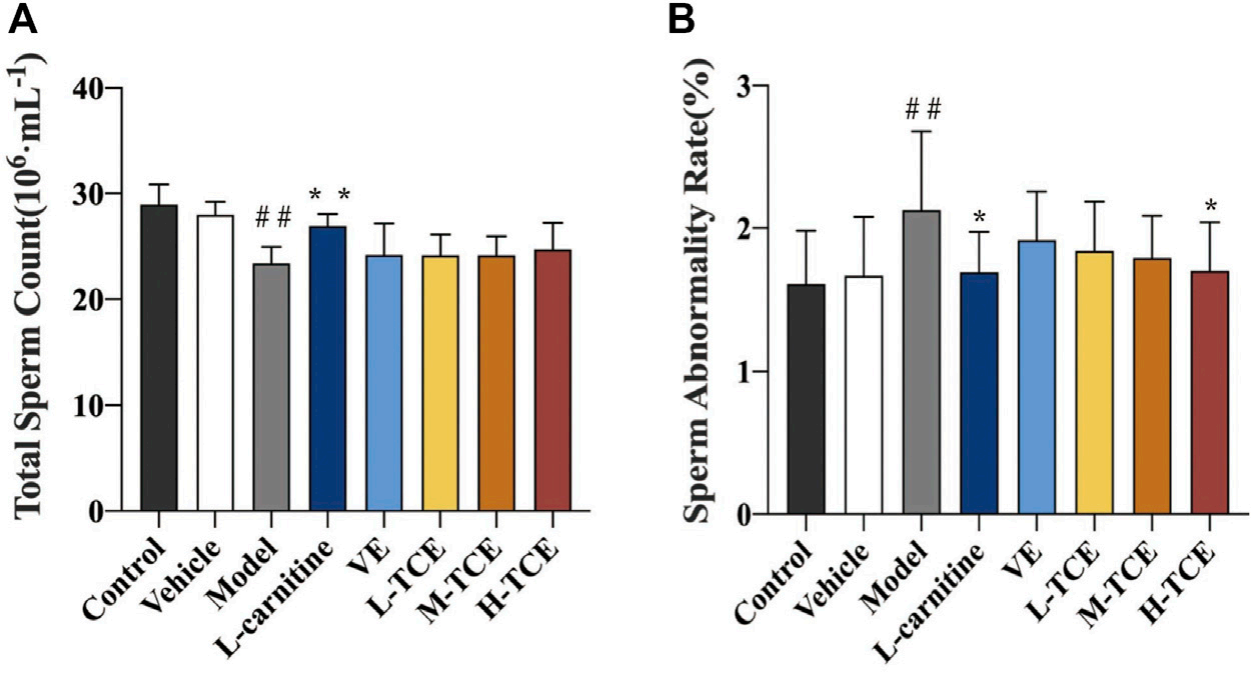
Changes in total sperm count and sperm abnormality rates in the different groups **(A)**. Total sperm count; **(B)**. Sperm abnormality rate; Means ± SD, *n* = 12; *p < .05, compared with the model group; **p < .01, compared with the model group; ^##^ *p* < .05, compared with the control group.

Significant alteration in the total sperm count and sperm abnormality rate was observed in the model group compared with the control group (*p* < .05), as confirmed by Figure 4. Results substantiated a significant decline in the sperm abnormality rate of the H-TCE group compared with the model group (*p* < .05).

#### Result of T, FSH, LH, and E2

**FIGURE 5:**
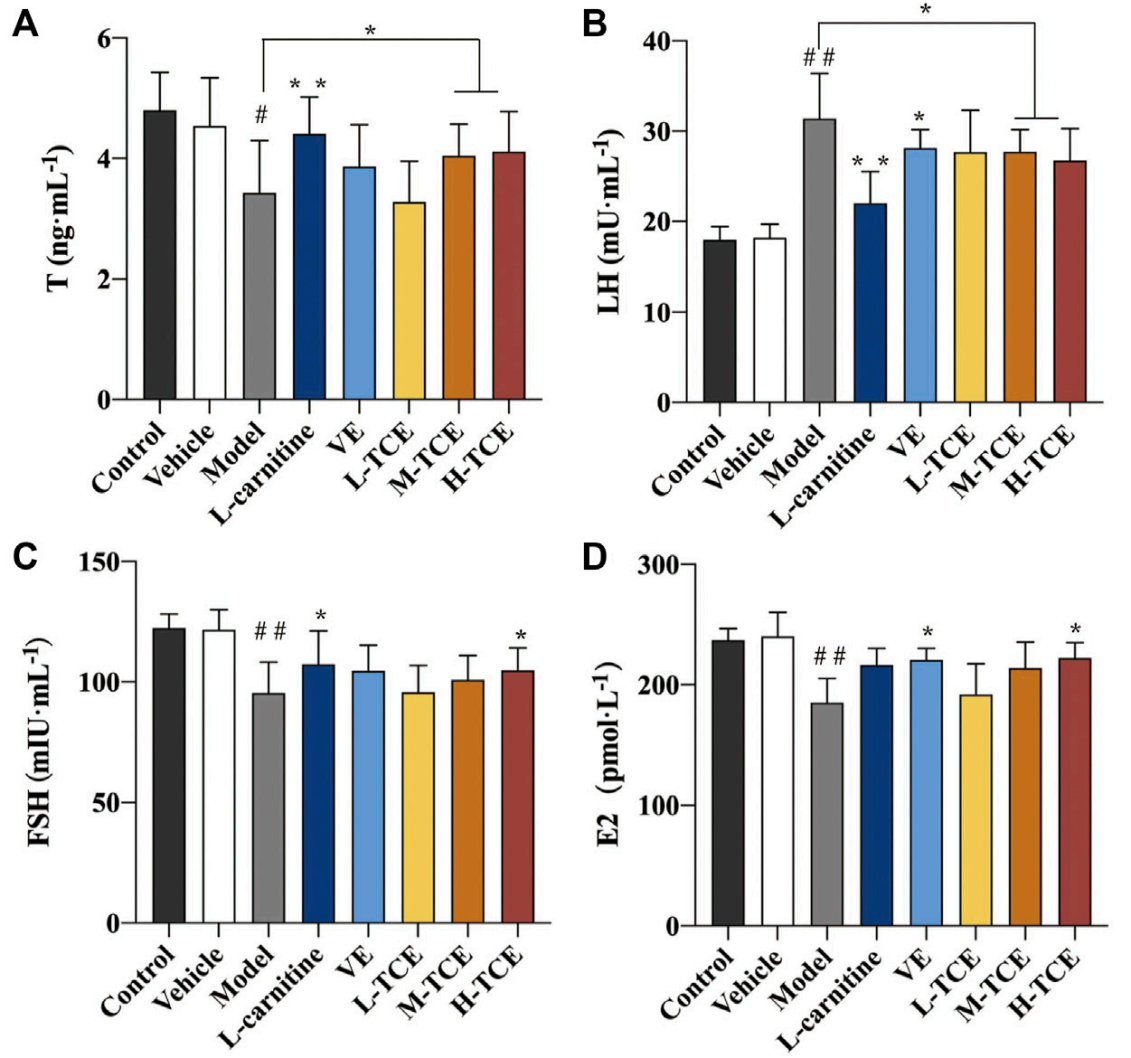
Changes in T, LH, FSH and E2 in the different groups **(A)**. T; **(B)**. LH; **(C)**. FSH; **(D)**. E2; Means ± SD, *n* = 12; *p < .05, compared with the model group; **p < .01, compared with the model group; ^##^ *p* < .05, compared with the control group.

Table 3 details the linear equations for the determination of sex hormone content. As indicated by Figure 5, induction with DEHP significantly reduced the serum levels of T, FSH and E2, whereas elevated the levels of LH (*p* < .05). Compared with the model group, the H-TCE group experienced a significant increase in serum T, FSH and E2 contents, while LH content was decreased (*p* < .05). The serum level of T and LH were also significantly changed in the M-TCE group relative to that in the model group (*p* < .05).

**TABLE 3:** Linear equation for the determination of sex hormone content.

	Linear equations	*R* ^2^	Linear range
T	y = 0.0572x + 0.1282	0.9944	0.75–24 (ng·mL^−1^)
LH	y = 0.0163x + 0.1089	0.9981	1.25–20 (mU·mL^−1^)
FSH	y = 0.0104x + 0.042	0.9905	2.5–80 (mU·mL^−1^)
E2	y = 0.0048x + 0.0017	0.9907	7.8–400 (pmol·L^−1^)

#### Results of AKP, ACP, LDH

**FIGURE 6:**
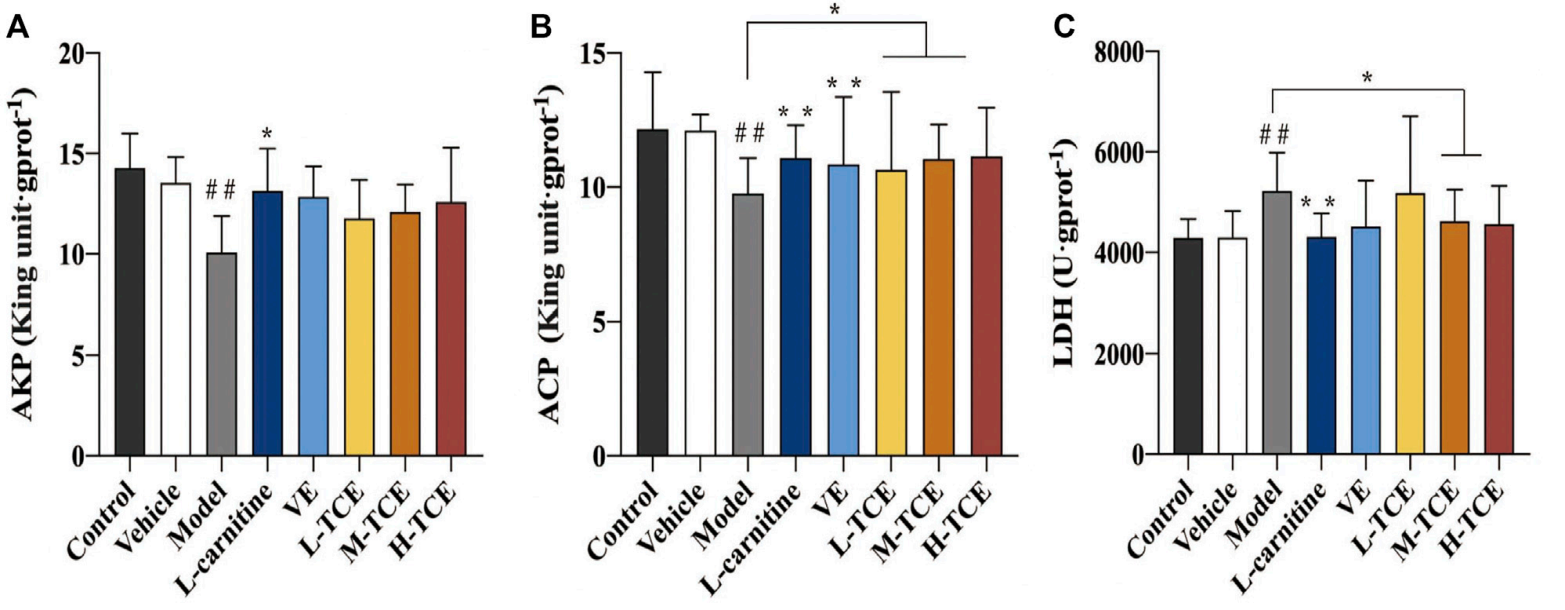
Changes in body AKP, ACP, LDH in the different groups **(A)**. AKP; **(B)**. ACP; **(C)**. LDH; Means ± SD, *n* = 12; *p < .05, compared with the model group; **p < .01, compared with the model group; ^##^ *p* < .05, compared with the control group.

The DEHP-induced mice revealed significantly lower levels of AKP and ACP, while higher levels of LDH (*p* < .05) in the testicular tissue homogenate (Figure 6). Compared with the control group, the ACP contents were significantly augmented in the M-TCE and H-TCE groups, whereas the LDH content significantly subsided, and there were significant differences in ACP in the L-TCE group (*p* < .05). Nonetheless, there was no significant difference in AKP but a dose relationship.

#### Result of ATPase Activity

**FIGURE 7:**
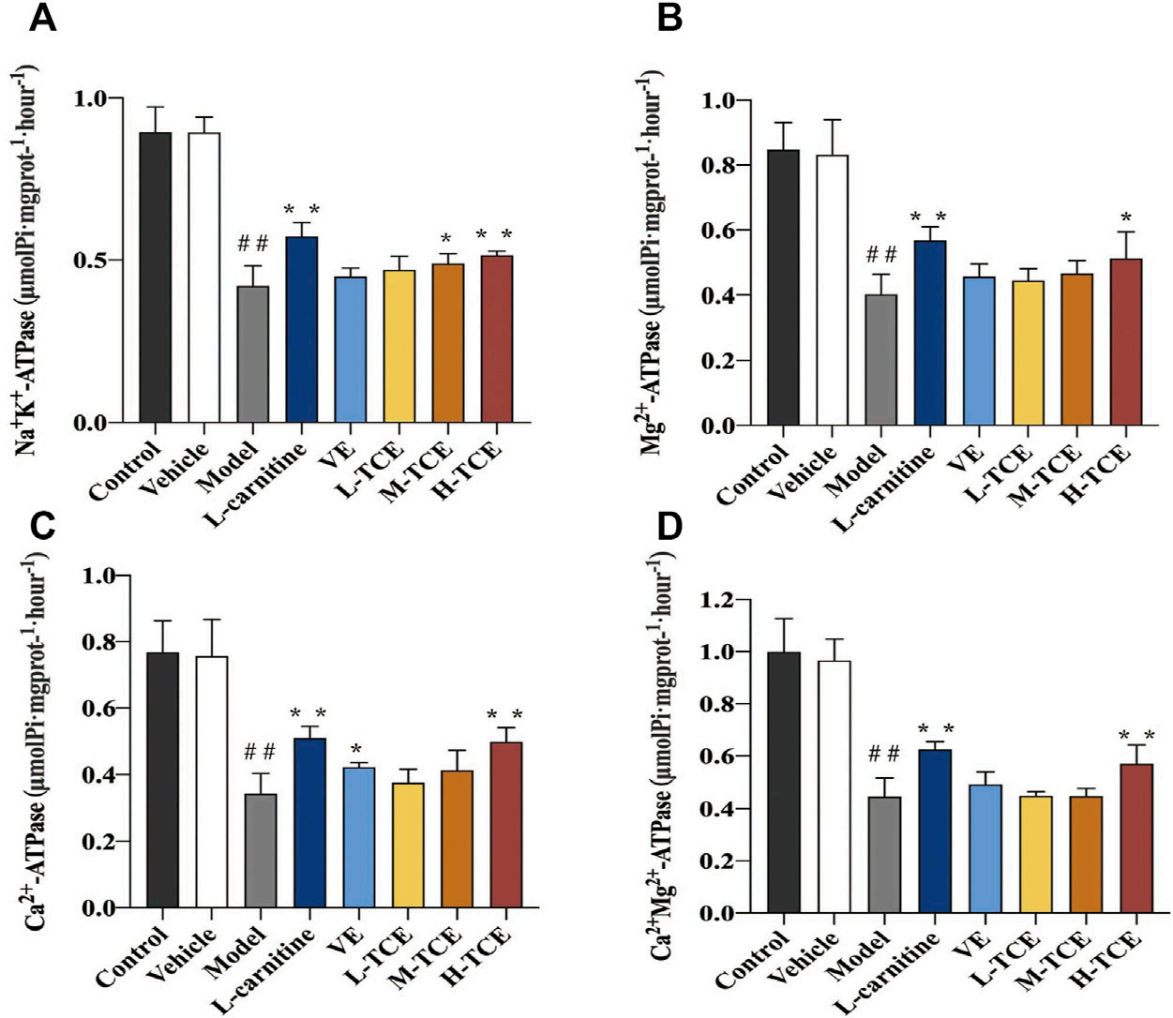
Changes in Na^+^K^+^, Mg^2+^, Ca^2+^, and Ca^2+^ Mg^2+^-ATPase in the different groups **(A)**. Na^+^K^+^-ATPase; **(B)**. Mg^2+^-ATPase; **(C)**. Ca^2+^-ATPase; **(D)**. Ca^2+^ Mg^2+^-ATPase. Means ± SD, *n* = 12; *p < .05, compared with the model group; **p < .01, compared with the model group; ^##^ *p* < .05, compared with the control group.

As shown in Figure 7, the mice induced with DEHP witnessed a significant decline in the testicular tissue homogenate levels for each ion channel activity (*p* < .05). In comparison to the control group, the Ca^2+^Mg^2+^-ATPase, Mg^2+^ -ATPase and Ca^2+^-ATPase levels were significantly increased in the H-TCE group, while in the M-TCE and H-TCE groups, Na^+^K^+^-ATPase was significantly increased (*p* < .05).

#### Results of SOD, MDA, GSH-Px, GSH, GR, and CAT

**FIGURE 8:**
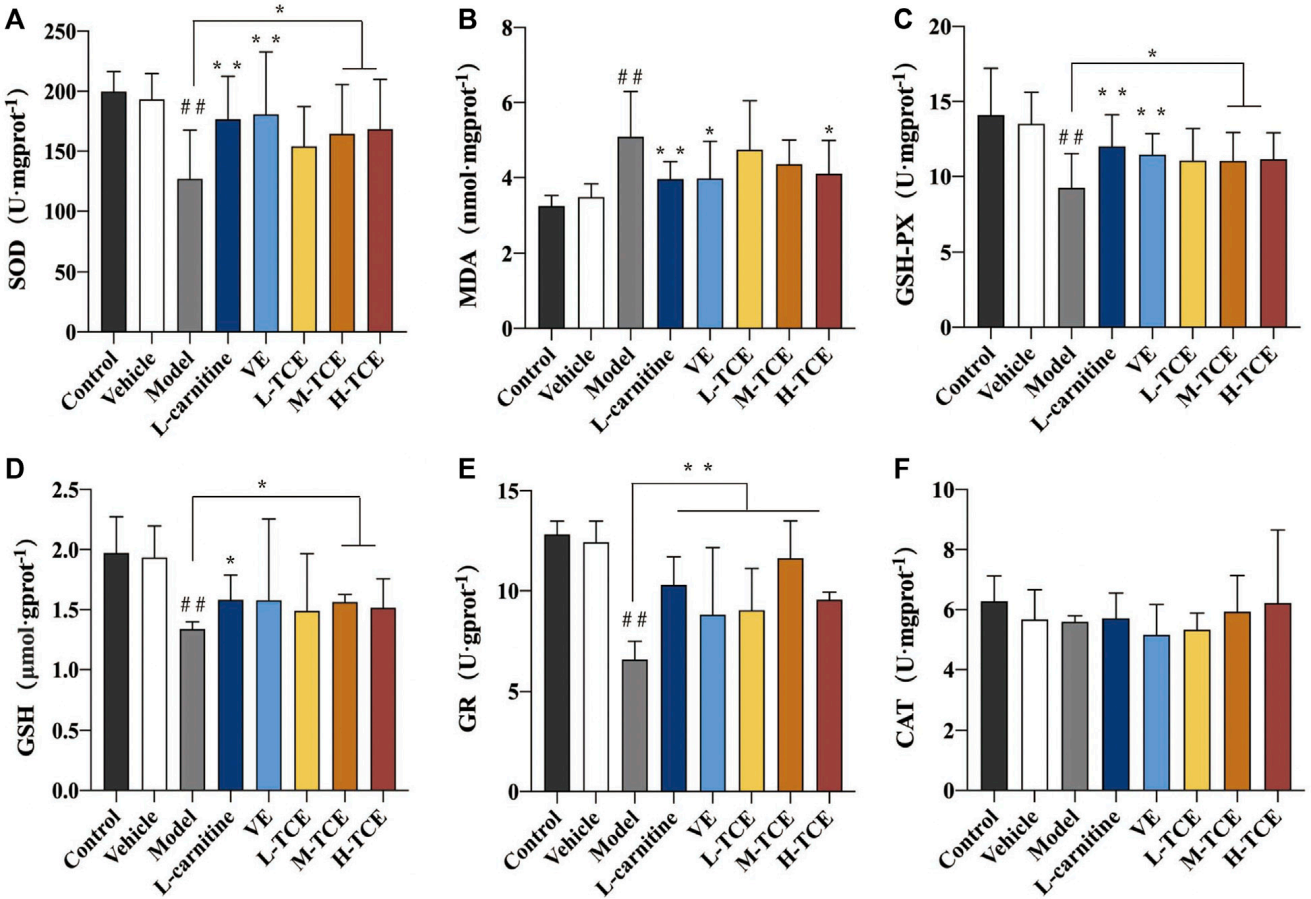
Changes in SOD, MDA, GSH-Px, GSH, GR and CAT in the different groups **(A)**. SOD; **(B)**. MDA; **(C)**. GSH-Px; **(D)**. GSH; **(E)**. GR; **(F)** CAT; Means ± SD, *n* = 12; *p < .05, compared with the model group; **p < .01, compared with the model group; ^##^ *p* < .05, compared with the control group.

Significant decrease in the activities of SOD, GSH-Px GSH, and the content of GR, and increase in MDA content (*p* < .05) were prominent in the testicular tissue homogenate of the DEHP-induced mice (Figure 8). Compared with the control group, the M-TCE and H-TCE groups corroborated significant escalation in the SOD, GSH-Px, and GSH activities, while the MDA content was significantly diminished (*p* < .05) H-TCE group. Moreover, the H-TCE, M-TCE, and L-TCE reflected significant up-regulation in the GR content (*p* < .01), but had no effect in CAT.

### Result of qRT-PCR Detection

**FIGURE 9:**
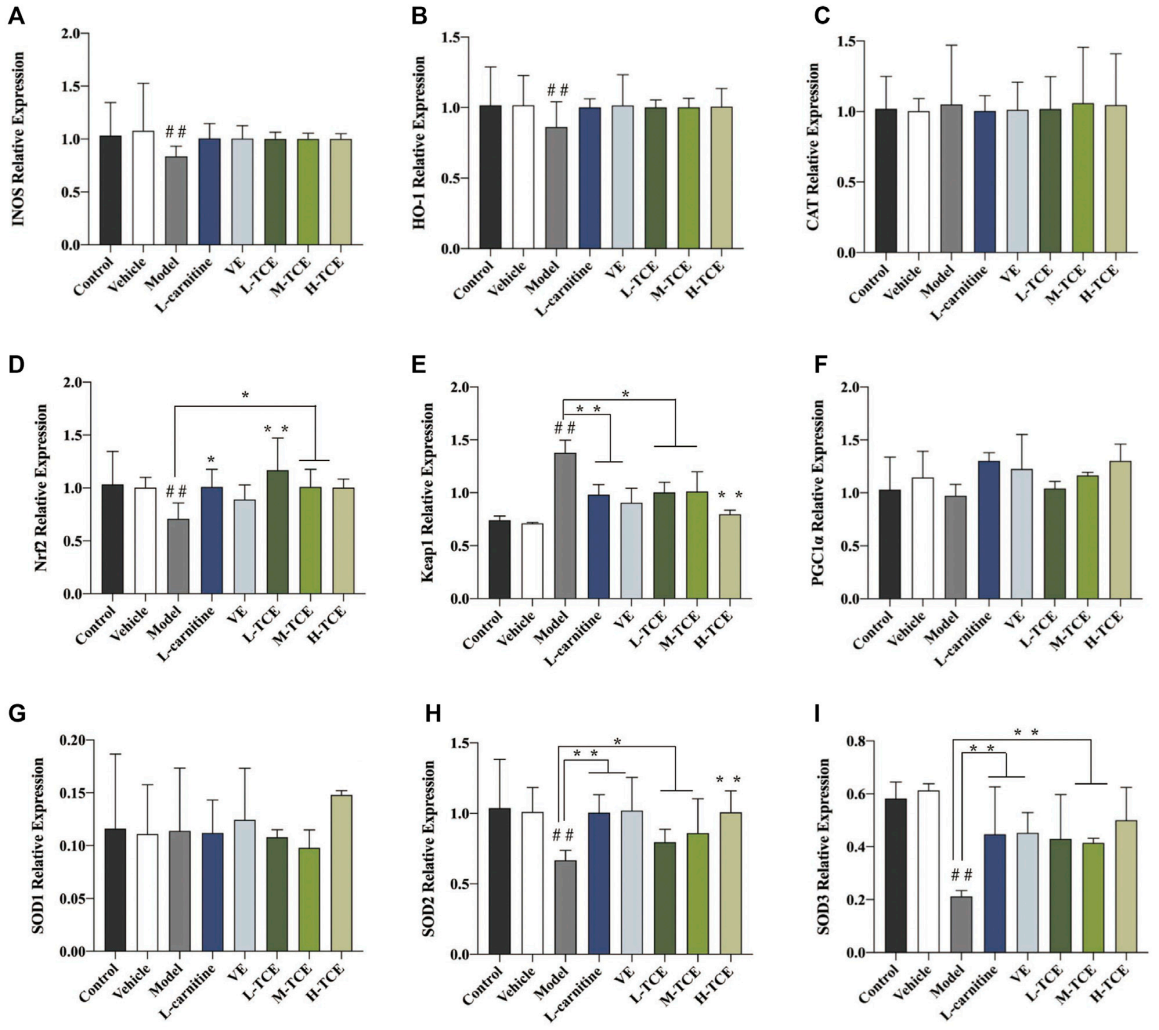
The relative expression levels of nine key oxidation-related genes. **(A)**. *INOS*; **(B)**. *HO-1*; **(C)**. *CAT*; **(D)**. *Nrf2*; **(E)**. *Keap1*; **(F)**. *PGC1α*; **(G)**. *SOD1*; **(H)**. *SOD2*; **(I)**. *SOD3*; Means ± SD; *n* = 6; *p < .05, compared with the model group; **p < .01, compared with the model group; ^##^ *p* < .05, compared with the control group.

**FIGURE 10:**
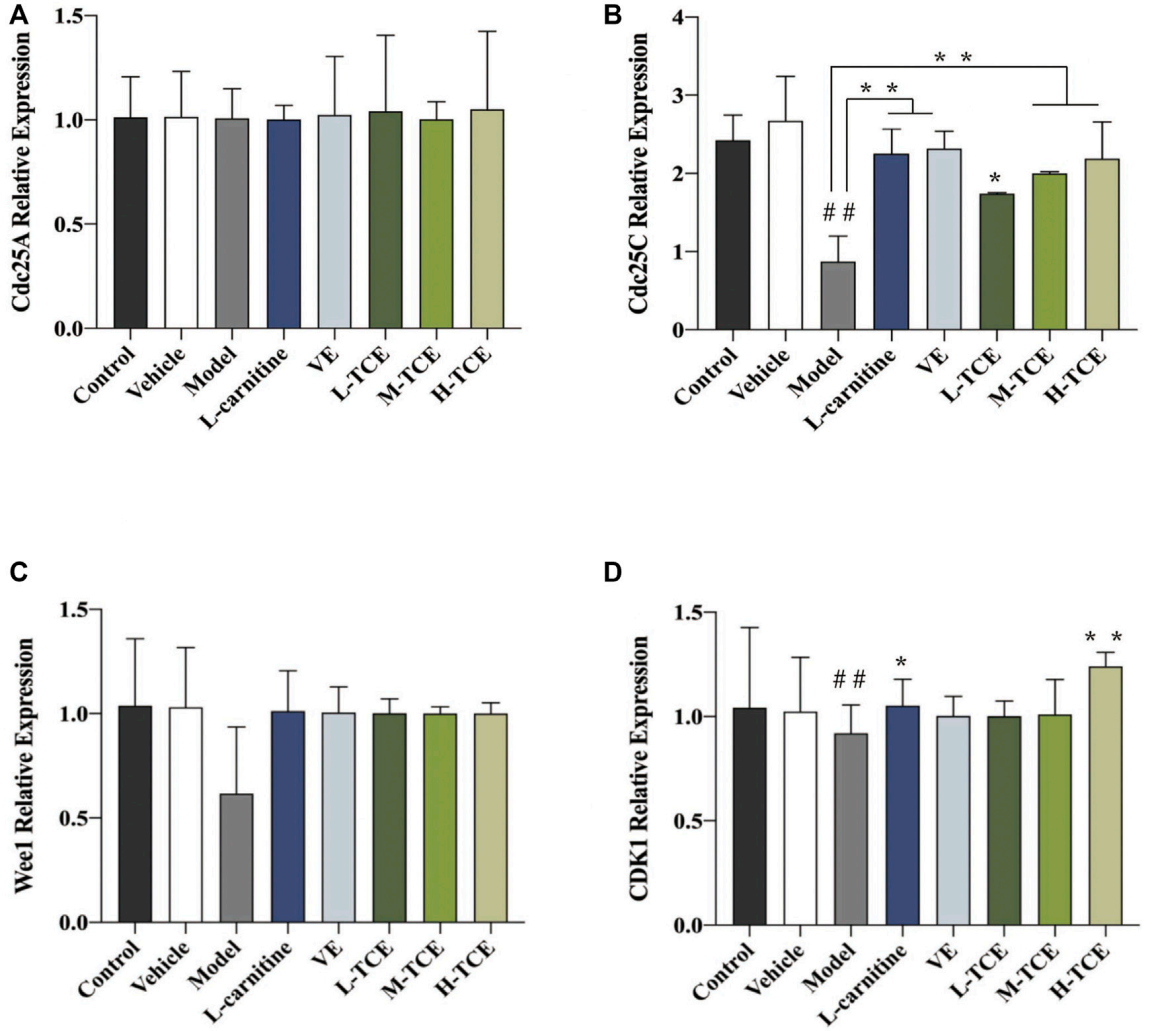
The relative expression levels of four key cell cycle control-related genes. **(A)**. *cdc25A*; **(B)**. *cdc25C*; **(C)**. *Wee1*; **(D)**. *CDK1*; Means ± SD; *n* = 6; *p < .05, compared with the model group; **p < .01, compared with the model group; ^##^ *p* < .05, compared with the control group.

**FIGURE 11:**
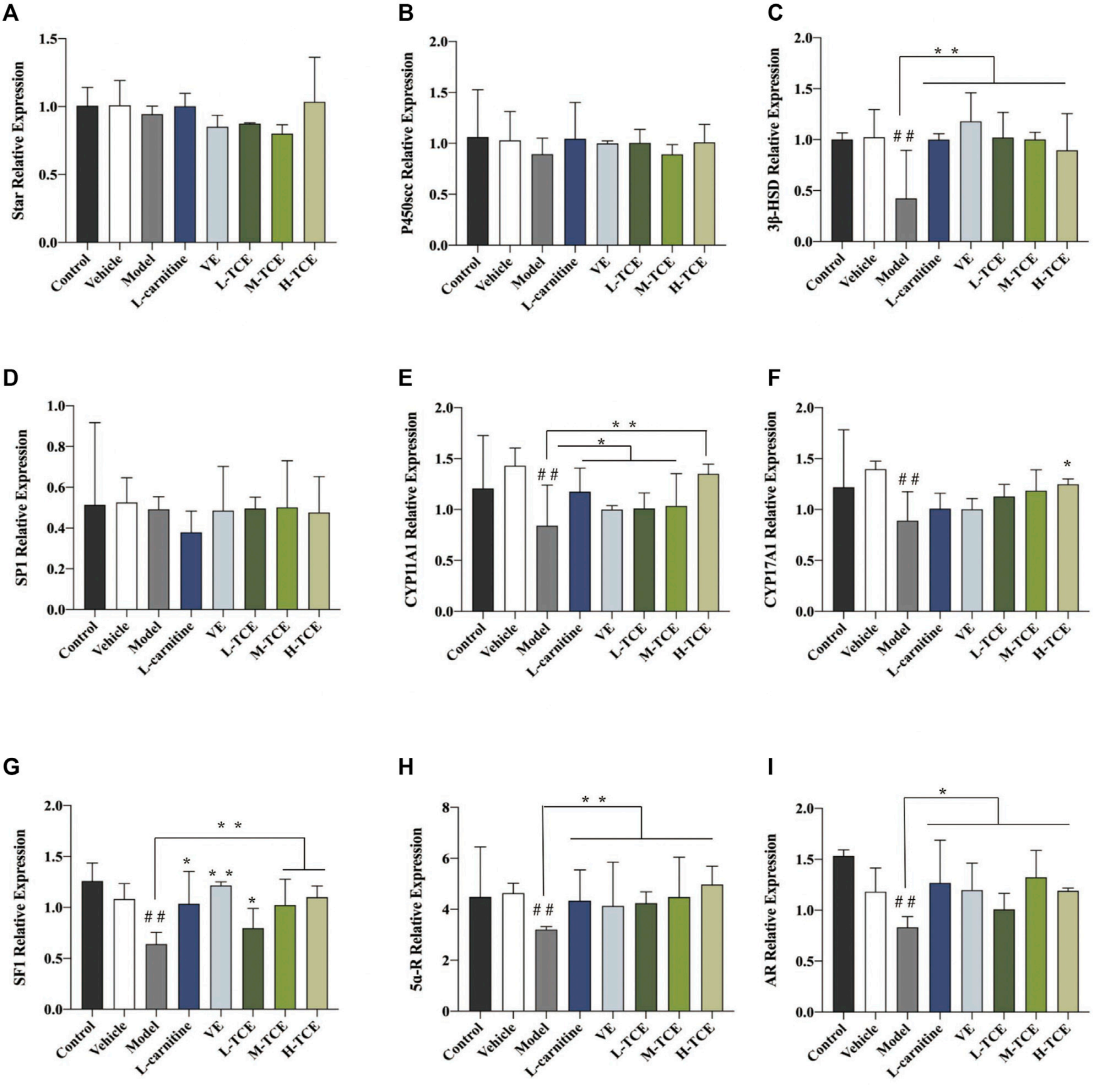
The relative expression levels of nine key steroidogenic-related genes. **(A)**. *star*; **(B)**. *P450scc*; **(C)**. *3β-HSD*; **(D)**. *SP1*; **(E)**. *CYP11A1*; **(F)**. *CYP17A1*; **(G)**. *SF1*; **(H)**. *5ɑ-R*; **(I)**. *AR*; Means ± SD; *n* = 6; *p < .05, compared with the model group; **p < .01, compared with the model group; ^##^ *p* < .05, compared with the control group.

The qRT-PCR determined the expression levels of nine key oxidation-related genes, four key cell cycle control-related genes and nine key steroidogenic-related genes (Figures 9–11). For the nine key oxidation-related genes, relative to the model group, the H-TCE, M-TCE, and L-TCE could significantly up-regulate the mRNA levels of *Nrf2, SOD2,* and *SOD3,* whereas significantly down-regulate the mRNA levels of *Keap1* (*p* < .05), but did not affect *INOS, HO-1, CAT, PGC1α,* and *SOD1.* Compared with the model group, for the four key testicular cell cycle control-related genes, the H-TCE, M-TCE, and L-TCE reflected significant up-regulation in the mRNA levels of *CDC25C*, and *CDK1* (*p* < .05); but had no effect on *CDC25A, Wee1*. For the nine key steroidogenic-related genes, compared with the model group, the H-TCE, M-TCE, and L-TCE could significantly up-regulate the mRNA levels of *CYP11A1,3β-HSD, 5ɑ-R, AR,* the M-TCE, and H-TCE reflected significant up-regulation in the mRNA levels of *SF1,* and significant up-regulation in the mRNA levels of *CYP17A1* in the H-TCE. Nonetheless, there was no significant difference in *SP1, P450scc* and *Star*.

### IHC Studies

**FIGURE 12:**
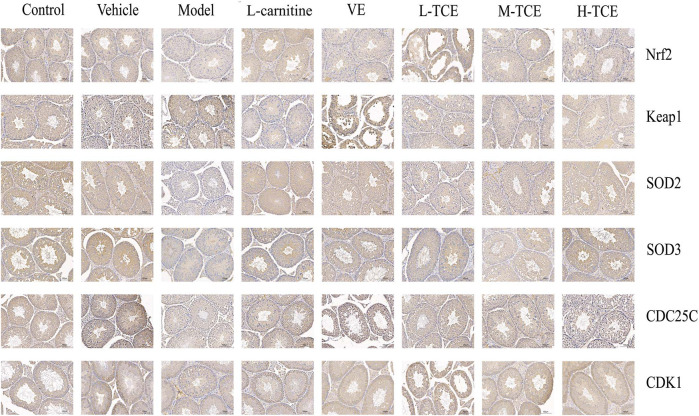
IHC analysis of the testis tissues. Immunopositive expression of Nrf2, Keap1, SOD2, SOD3, CDC25C, and CDK1 was observed. Original magnification, ×200.

**FIGURE 13:**
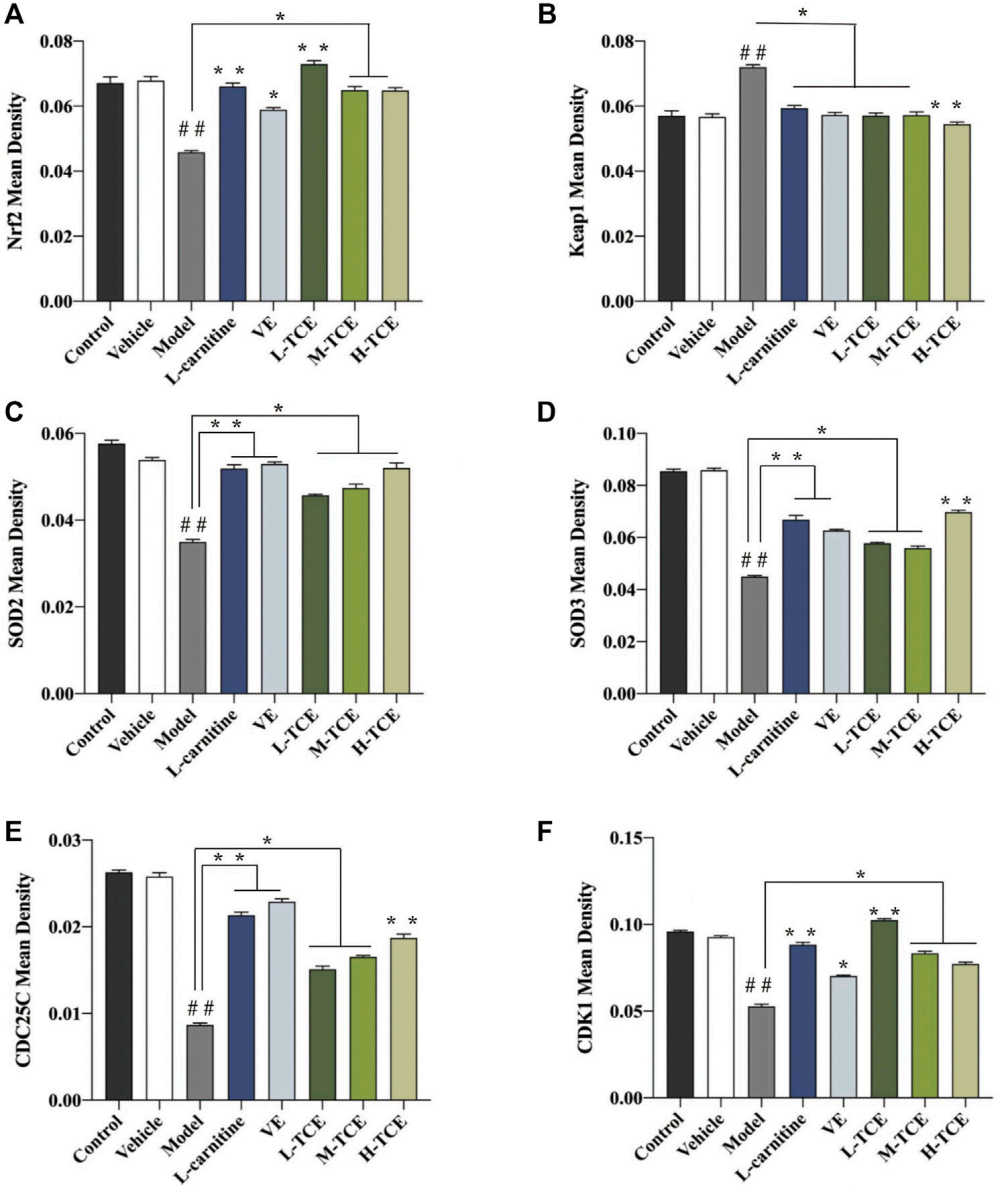
The mean density of six antibodies. **(A)**. Nrf2; **(B)**. Keap1; **(C)**. SOD2; **(D)**. SOD3; **(E)**. CDC25C; **(F)**. CDK1; Means ± SD; *n* = 6; *p < .05, compared with the model group; **p < .01, compared with the model group; ^##^ *p* < .05, compared with the control group.

A brownish-yellow area represented a positive result, indicating antigen-antibody binding on germ cells. The mean density is computed as the ratio of the cumulative optical density value (IOD) to the pixel area of the tissue (AREA) for each positive photograph, and the immunohistochemical results can be interpreted by analyzing mean density. The lower mean density of Nrf2, SOD2, SOD3, CDC25C, and CDK1 proteins in the DEHP group signified weaker immunopositive staining and lower protein expression for each protein following DEHP induction. The higher mean density of Keap1 proteins in the DEHP group signified higher protein expression following DEHP induction (*p* < .01). However, in the TCE group, the testicular tissue revealed a trend of significantly enhanced positive responses to Nrf2, SOD2, SOD3, CDC25C, and CDK1 proteins with significantly higher protein expression and a trend of significantly reduced positive responses to Keap1 proteins with significantly lower protein expression (Figures 12, 13).

## Discussion

### TCE-Mediated Alleviation of the Oxidative Stress Caused by DEHP

The findings of this study suggested that DEHP considerably increased MDA content in testicular serum, while GSH, GSH-Px, SOD vitality, and GR content were significantly reduced, indicating that DEHP staining in testicular tissue failed to remove reactive oxygen free radicals effectively. This resulted in excessive accumulation of oxygen free radicals, which, in turn, triggered lipid peroxidation, ultimately leading to cell damage. Significantly affecting the testicular tissue oxidation and antioxidant enzyme content, H-TCE enabled the repair of the DEHP-induced oxidative damage of testicular tissue. The effect of TCE in modifying the activity of SOD and other oxidation indicators encouraged us to study the activity of related genes by qRT-PCR. Results emphasized TCE-mediated significant increase in the expression levels of *Nrf2, SOD2,* and *SOD3, a* significant decrease in the expression levels of *Keap1*. IHC further substantiated this finding at the protein level. Keap1-Nrf2 pathway plays a crucial role in antioxidant activity. In the resting state, *Nrf2* binds to Keap1 in the cytoplasm and is inactive. When the body is in a state of oxidative stress, *Nrf2* is uncoupled with *Keap1* and transferred into the nucleus, where it binds with antioxidant reaction elements (ARE) to activate the expression of downstream antioxidant protein genes to reduce oxidative damage (Papaiahgari et al., 2007; Ma, 2013). TCE may stimulate oxidative degradation of *Keap1* in testicular tissue cytoplasm, promote dissociation of Keap1-Nrf2 coupling, increase nuclear translocation and expression of *Nrf2*, and then activate transcription of antioxidant enzymes such as SOD, GSH, and GSH-Px downstream to enhance antioxidant damage ability and resist testicular injury (Uruno and Motohashi, 2011). The increase of SOD enzyme activity can reduce the ROS generation and regulate the level of O^2−^; thus, minimizing the detrimental effect of oxidative stress and maintaining normal tissue function (Schram et al., 2019). TCE’s better anti-oxidative activity of the reproductive system may be realized by up-regulating SOD family expression and Keap1-Nrf2 signaling pathway.

The phenolic compounds may be the pharmacologically active ingredients responsible for the antioxidant activity of TCE. Phenolic compounds are a special type of secondary metabolite, with phenolic radicals having a lower electron reduction potential than that of oxygen radicals (Bors et al., 1990; Grace, 2005) and the capacity to quench radicals by forming resonance-stabilized phenoxy groups (Rice-Evans et al., 1996; Bors and Michel, 2002). Thus, by scavenging the intermediates of reactive oxygen species, these phenolic compounds restrict the promotion of further oxidation reactions (Grace, 2005). By maintaining the homeostasis between oxidants and antioxidants, they play a vital role in combating oxidative stress in humans (Manach et al., 2005; Dudonné et al., 2009).

### TCE Improved the Energy Metabolism

ATPase is a widely present key enzyme in testicular energy metabolism, affecting energy production, transfer, and utilization. It also regulates normal membrane potential changes in cell membranes, thereby maintaining the stability of the internal environment. The reductions of ATPase and reduced ATP production exert a crucial impact on tissue damage (Pivovarova et al., 1992). This investigation revealed a significant reduction in ATPase activity following DEHP induction, and this indicated that DEHP-induced inhibition of ATPase activity affects the testicular spermatogenic cells to carry out energy conversion. This, in turn, impaired the development of the testis and epididymides, causing damage to the spermatogenic cells and affecting the spermatogenic process. Alteration in testicular and epididymides organ indexes and sperm-related parameters further substantiated these findings. In this study, DEHP-induced inhibition of gonadal organ development, a decline in organ coefficients of testes and epididymides, and an increase in sperm aberration rate all confirmed the abnormal energy metabolic process of the reproductive system. H-TCE was able to restore enzyme activities and organ indices, and sperm-related parameters to some extent, indicating that TCE reinstated the energy metabolic process of the reproductive system to a certain degree.

### TCE Improved the Testicular Function

Moreover, changes in sex hormones and testicular marker enzymes reflect modification in testicular function. The major physiological function of T, mostly synthesized and secreted by testicular mesenchymal cells, is to maintain normal spermatogenesis and promote the development and maturation of the testicular parenchymal organs (Han et al., 2021). The hypothalamic-pituitary-testicular axis (HPTA) is primarily engaged in regulating the synthesis and secretion of testosterone, which may also be regulated by feedback from LH and FSH (Recabarren et al., 2013; Ha et al., 2016). The entry of the capital products of T into the reproductive system following DEHP stimulation may damage mesenchymal cells through oxidative damage and other pathways, resulting in a decrease in T levels. It may also produce large amounts of estrogen, interfering with the normal function of HPTA and affecting the secretion of LH and thus testosterone. This diminishes the serum testosterone levels in mice, decreases its binding to androgen receptors in spermatogenic cells, thereby inhibiting spermatogenesis.

AKP activity is linked to the division of testicular spermatogenic cells at all stages, as well as with the energy transport of glucose. ACP is most abundant in the cytoplasm of testicular supporting cells and is involved in spermatogenic cell synthesis and phagocytosis. It plays a vital role in protein synthesis and supports normal physiological metabolism and function. Changes in its activity are directly related to the degeneration of the germinal epithelium (Zheng et al., 2010). LDH is a testis-specific marker enzyme, closely linked with the germinal process, and changes in its activity can respond characteristically to the lethal effects of chemical toxicants on the testis (Hodgen and Sherins, 1973; Yang et al., 2010). Interfering with glycolysis and aerobic respiration in germinal cells, DEHP can lead to an inadequate supply of energy, affecting spermatogenesis and maturation. H-TCE significantly altered ACP and LDH, reversing the effects triggered by DEHP and facilitating energy transfer to testicular tissue.

Estimation of the cell cycle control-related genes claimed a significant decrease in *CDK1* and *CDC25C* levels following DEHP induction. *CDK1* is a nonredundant cyclin-dependent kinase with an essential role in mitosis (Vassilev et al., 2006). Studies have established a correlation of the activation of this kinase with cell death of postmitotic neurons in brain development and disease (Yuan et al., 2008). *CDC25C,* a dual-specificity protein phosphatase, monitors the entry of cells into mitosis by dephosphorylating the protein kinase Cdc2 (Peng et al., 1997). Phosphorylation on serine 216 is observed throughout interphase but not during mitosis (Peng et al., 1998). The present study documented that exposure to DEHP impaired the CDC25C-CDK1 interaction during mitosis. This decelerates the metabolism of spermatogenic cells, curtailing sperm growth and maturation. The M-TCE and H-TCE facilitated a significant upsurge in the expression of *CDK1*, *CDC25C,* proposing the role of TCE in promoting CDC25C-CDK1 interaction, which in turn stimulates spermatogenic cell proliferation, testicular development, and sperm development and maturation.

Testosterone in the Leydig cells of synthetic process for combining with the status of cholesterol in the blood after receptor transport into the Leydig cell cytoplasm, with the help of STAR, and so on factor into the mitochondrial membrane, on P450SCC, mitochondrial membrane cholesterol cracking can become pregnenolone, is the first step on the testosterone generated in this reaction, It is also the rate-limiting step of testosterone production (Agarwal and Auchus, 2005). After the synthesis of pregnenolone, it is transferred to the smooth endoplasmic reticulum by diffusion, where it is rapidly digested into progesterone by 3β-HSD. 3β-HSD is one of the key enzymes of the steroid hormone synthesis pathway and is expressed in testicular interstitial cells. It is recognized as a marker of testicular interstitial cells. Its activity is also an important indicator to measure the ability of interstitial cells to synthesize testosterone, which is the basic step of all bioactive steroid hormone synthesis (Trant et al., 1990; Sawicki et al., 1999; Zylka et al., 2008). CYP11A1 and CYP17A1 are important enzymes in the transformation of pregnenolone to androgen (Sushko et al., 2012; Bloem et al., 2013). SF1 affects testosterone androgen synthesis by interfering with the signal transduction pathway of steroid hormone synthesis and interfering with the expression of genes and proteins related to cholesterol transport or androgen synthesis. 5α-R is a specific enzyme that catalyzes the transformation of testosterone into dihydrotestosterone (DHT). The high expression of DHT can promote the development of organs such as testis and epididymides, which is consistent with the results of organ coefficient measurement. Its activity also affects the development and maturation of sperm. AR is the only receptor for androgen binding, and its higher expression level indicates that the number of androgen binding receptors is increased to promote androgen activity (Poletti and Martini, 1999; Wang et al., 2019). Compared with the control group, the relative expression levels of *3β-HSD, CYP11A1, CYP17A1, AR,* and *5ɑ -R, SP1* were decreased. Compared with the model group, 900 mg kg^−1^ generally increased the relative expression levels of the above genes.

## Conclusion

Our results suggest that M-TCE and H-TCE was able to alleviate DEHP-induced reproductive system damage in male mice by improving testicular histopathology, repairing testicular function, and reducing oxidative stress damage. The differences in indicators among the groups showed that TCE significantly improved the anogenital distance and the organ indexes of the epididymides and testes. It also significantly reduced the extent of varicocele and interstitial cell lesions compared to the model group. H-TCE reduced the sperm abnormality rate, increased the levels of sex hormones, Na^+^K^+^ and Mg^2+^, Ca^2+^-ATPase enzyme activity, antioxidant enzyme vitality, along a significant decrease in LH and MDA contents. The levels of testicular marker enzymes ACP and LDH were significantly augmented by both M-TCE and H-TCE. qRT-PCR and IHC results indicated that the mechanistic approach behind the protective effects of TCE might be attributed to the oxidation-related Keap1-Nrf2 pathway, SODs enzyme, the cell cycle control-related CDC25C-CDK1 pathway, and the steroidogenic-related pathway. Thus, in summary, our results confirmed the beneficial effects of *Trichilia catigua* on male mice reproduction. The mechanisms of the beneficial effects are complex and ambiguous but may include the modulation of antioxidant, energy metabolism, and testicular function.

In this study, the mechanism of TCE against oxidative damage in the reproductive system was preliminarily discussed. However, as the mechanism of testicular tissue function is complex and changeable, this mechanism is only one of the possible ways. Subsequent studies can explore the inhibition of mitochondrial damage and apoptosis to study the mechanism of TCE in a more systematic and in-depth way.

## Data Availability

The original contributions presented in the study are included in the article/Supplementary Material, further inquiries can be directed to the corresponding authors.

## Abbreviations

ACP, acid phosphatase; AKP, alkaline phosphatase; BTB, blood-testis barrier; CAT, catalase; DEHP, di-(2-ethylhexyl) phthalate; E2, estradiol; FSH, follicle-stimulating hormone; GR, glutathione reductases; GSH, reduced glutathione; GSH-Px, glutathione peroxidase; i.g., intragastric administration; IHC, immunohistochemical; LD0, 0% lethal dose; LD50, median lethal dose; LD100, 100% lethal dose; LDH, lactate dehydrogenase; LH, luteinizing hormone; MDA, malondialdehyde; MTD, maximum tolerated dose; SOD, superoxide dismutase; T, testosterone; TCE, *Trichilia catigua* A. Juss. extract; qRT-PCR, quantitative real-time PCR.
