# Supplementary material for: The Protective Effect of Trichilia catigua A. Juss. on DEHP-Induced Reproductive System Damage in Male Mice
Source: Front Pharmacol. 2022 Feb 3;13:832789. doi: 10.3389/fphar.2022.832789 (PMC8853101; doi:10.3389/fphar.2022.832789)
Supplement: Supplementary file 1 [file DataSheet1.docx]

Supplementary Material

**Determination of DEHP molding conditions**

**Methods**

# 1 Animal grouping and dosing

The preparation of the sample solution: 25.0 g of DEHP was accurately weighed in a 100 mL volumetric flask, corn oil was used as co-solvent, and the volume of DEHP solution was fixed at a concentration of 250 mg·mL^–1^ for the high-dose group, while the three low-dose groups were diluted to the corresponding concentration with corn oil in steps of 0.02 mL·g^–1^, i.e., the doses administered were 5.0, 2.5, 1.25 and 0.625 mg·g^–1^, respectively.

Thirty male icr mice (18–22 g) were randomly and evenly divided into five groups: the control group (distilled water. i.g.) and different dosed administration groups (0.625, 1.25, 2.50, 5.0 mg·g^–1^ DEHP. i.g.). The animals were observed for their activity, hair, and food intake, and their body weight was weighed and recorded daily during the experiment. At the end of the experiment, the intact testes of the mice were removed and the molding condition was observed.

# 2 Testicular pathological analysis

The testes were obtained after whole-body perfusion of a 7-day-old male mouse with 4% paraformaldehyde. For operating instructions, please refer to section 2.6.3

# Result

In mice, the effects of various DEHP doses on testicular tissue were studied. There were numerous and closely spaced varicose tubules in the control group. The epithelium of the tubules consisted of a complex epithelium of spermatogenic cells and supporting cells having normal cell morphology and closely spaced cells. Further, long spindle-shaped spermatozoa were observed in the lumen of the tubules. Compared with the control group, with the increasing doses of DEHP, atrophy of the varicocele, a gradual decrease in epithelial cells, and necrosis of the germ cells were observed. When 5 mg·g^–1^ DEHP was administered, widespread dilatation of the germinal tubules, atrophy of the germinal tubules, swelling of the germinal cells, loosening of the cytoplasm (yellow arrow), necrosis and loss of the epithelial cells, and consolidation of the nuclei with deep staining or fragmentation (green arrow) were seen. The dose of DEHP was judged to be too high, so 2.5 mg·g^–1^ DEHP was chosen as the dose to be used for reproductive system damage in subsequent experiments (Fig. S1).

**BTB integrity check**

**Methods**

# 1 Animal grouping and dosing

The solution was prepared as directed in 2.5. Twenty male ICR mice (18–22 g) were divided into four equal groups (n = 5): Control (distilled water), model (2.5 mg·g^–1^ DEHP daily for 7 days, i.g.), VE (DEHP for 7 days then 100 mg·kg^–1^ VE, i.g.), H-TCE (DEHP for 7 days then 900 mg·kg^–1^ TCE, i.g.). Treatment was continued for 28 days.

# 2 BTB integrity check

At the end of the administration, the mice were anesthetized and exposed by dissection. The EZ-Link sulfo-NHS-LC-Biotin 50µL was injected with a BD syringe under the white membrane of the mouse testis, and the testis was returned. The abdominal cavity was closed for 30 minutes, then the abdominal cavity was reopened, and the testicular tissue was removed and prepared into a frozen section of 10µm thickness. Sections were fixed with 4% paraformaldehyde, washed with PBST for 3 times, and sealed with PBS(1x) containing 15% goat serum and 1%BSA for 30min. AlexaFluor 568 streptavidin working solution was incubated at room temperature for 2h. The tablets were sealed with anti-fluorescence quenching tablets containing DAPI, and the integrity of BTB in different groups was observed by fluorescence microscope (Canon PowerShot A95).

# qRT-PCR detection of gene expression

For operating instructions, please refer to section 2.7. The primer sequences for mice *Gapdh* (the housekeeping gene), *Occludin, ZO-1* are detailed in Table 1.

# Discussion

BTB is a special structure existing between Sertoli cells (SC)of the testis. SC are the main cells forming BTB, and various connective structures formed between SC are the structural basis of BTB function. It is also a key link in regulating the permeability of BTB (Wu *et al.*, 2020; Mruk *et al.*, 2015). When the body is subjected to external stimuli such as oxidative stress and ischemia, the integrity of BTB is destroyed, and barrier function is dysfunctional, leading to spermatogenesis disorder and loss of spermatogonial cells (Meroni *et al.*, 2019). ZO-1 and Occludin are the main structural proteins that form tight junctions, and they play a barrier function by forming complexes, which play a crucial role in maintaining the integrity and normal opening and closing of BTB (Su *et al.*, 2020; Feldman *et al.*, 2020; Müller *et al.*, 2005).

# Result

The integrity of BTB in different groups was observed. In the control group, the fluorescence was concentrated near the white membrane, indicating that the BTB was in good integrity. In the model group, there was strong fluorescence around and in the white membrane, which indicated that the BTB had great permeability and poor integrity. Both VE group and H-TCE group had certain fluorescence signal in the white membrane structure, indicating that the integrity of BTB was damaged to some extent, and the integrity of H-TCE was better than that of VE group. (Fig. S2).

The qRT-PCR determined the expression levels of 2 key BTB integrity-related genes (Fig. S3). Compared with the model group, the M-TCE, and L-TCE could significantly up-regulate the mRNA levels of *Occludin and ZO-1* (*P < 0.05*).

# Reference styles

Feldman, G. J., Mullin, J. M., & Ryan, M. P. (2005). Occludin: structure, function and regulation. Advanced drug delivery reviews, 57(6), 883–917. <https://doi.org/10.1016/j.addr.2005.01.009>

Meroni, S. B., Galardo, M. N., Rindone, G., Gorga, A., Riera, M. F., & Cigorraga, S. B. (2019). Molecular Mechanisms and Signaling Pathways Involved in Sertoli Cell Proliferation. Frontiers in endocrinology, 10, 224. <https://doi.org/10.3389/fendo.2019.00224>

Mruk, D. D., & Cheng, C. Y. (2015). The Mammalian Blood-Testis Barrier: Its Biology and Regulation. Endocrine reviews, 36(5), 564–591. <https://doi.org/10.1210/er.2014-1101>

Müller, S. L., Portwich, M., Schmidt, A., Utepbergenov, D. I., Huber, O., Blasig, I. E., & Krause, G. (2005). The tight junction protein occludin and the adherens junction protein alpha-catenin share a common interaction mechanism with ZO-1. The Journal of biological chemistry, 280(5), 3747–3756. <https://doi.org/10.1074/jbc.M411365200>

Su, L., Wang, Z., Xie, S., Hu, D., Cheng, Y. C., Mruk, D. D., & Guan, Y. (2020). Testin regulates the blood-testis barrier via disturbing occludin/ZO-1 association and actin organization. Journal of cellular physiology, 235(9), 6127–6138. <https://doi.org/10.1002/jcp.29541>

Wu, S., Yan, M., Ge, R., & Cheng, C. Y. (2020). Crosstalk between Sertoli and Germ Cells in Male Fertility. Trends in molecular medicine, 26(2), 215–231. <https://doi.org/10.1016/j.molmed.2019.09.006>


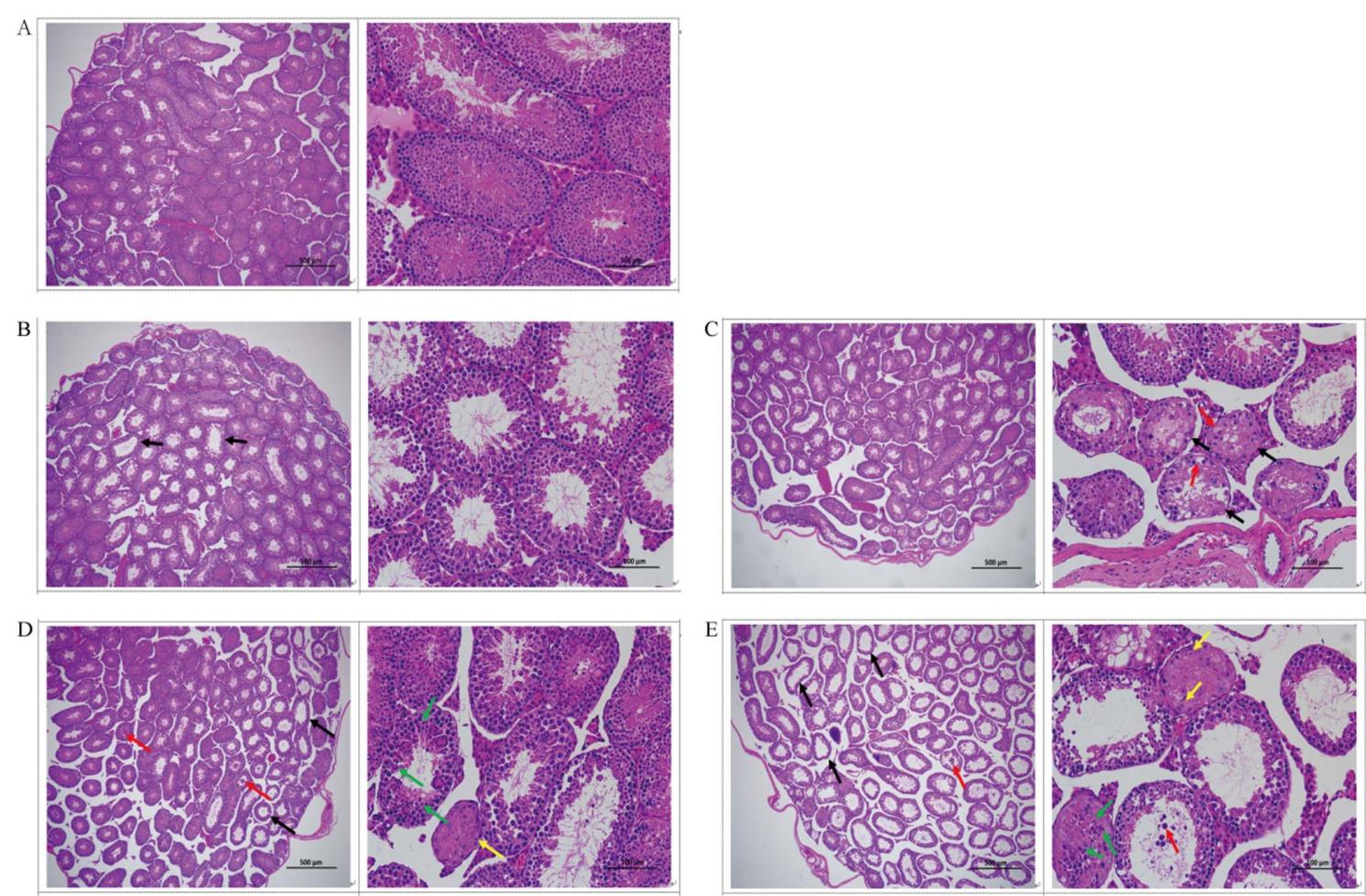


S1. Changes in testicular pathological analysis in the different groups A. Control group; B.0.625 mg·g^–1^ DEHP group; C. 1.25 mg·g^–1^ DEHP group; D. 2.5 mg·g^–1^ DEHP group; E. 5.0 mg·g^–1^ DEHP group; (x40, x200)
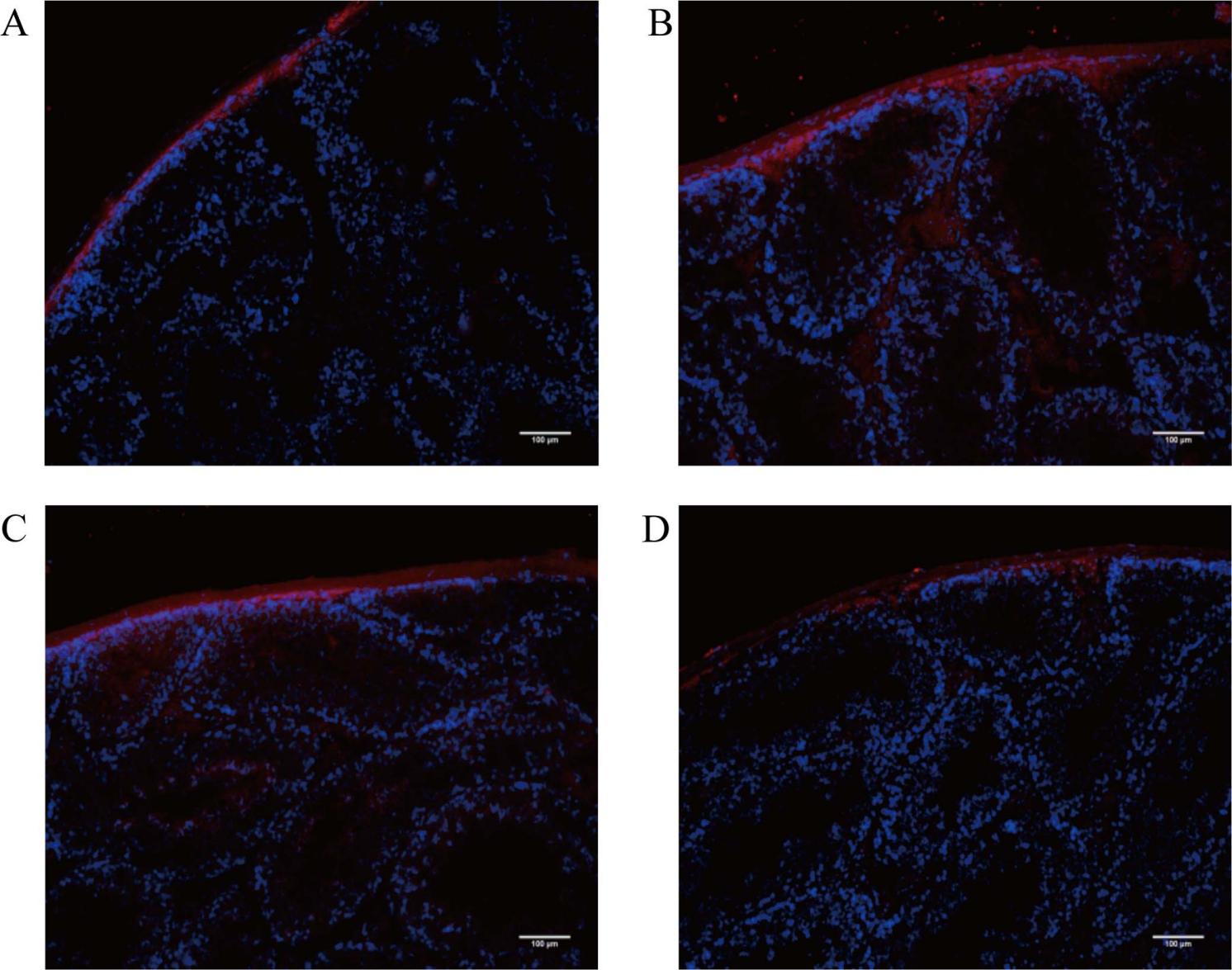


S2. Changes in the integrity of BTB in different groups A. Control group; B. Model group; C.VE group; D. H-TCE group; (x100)


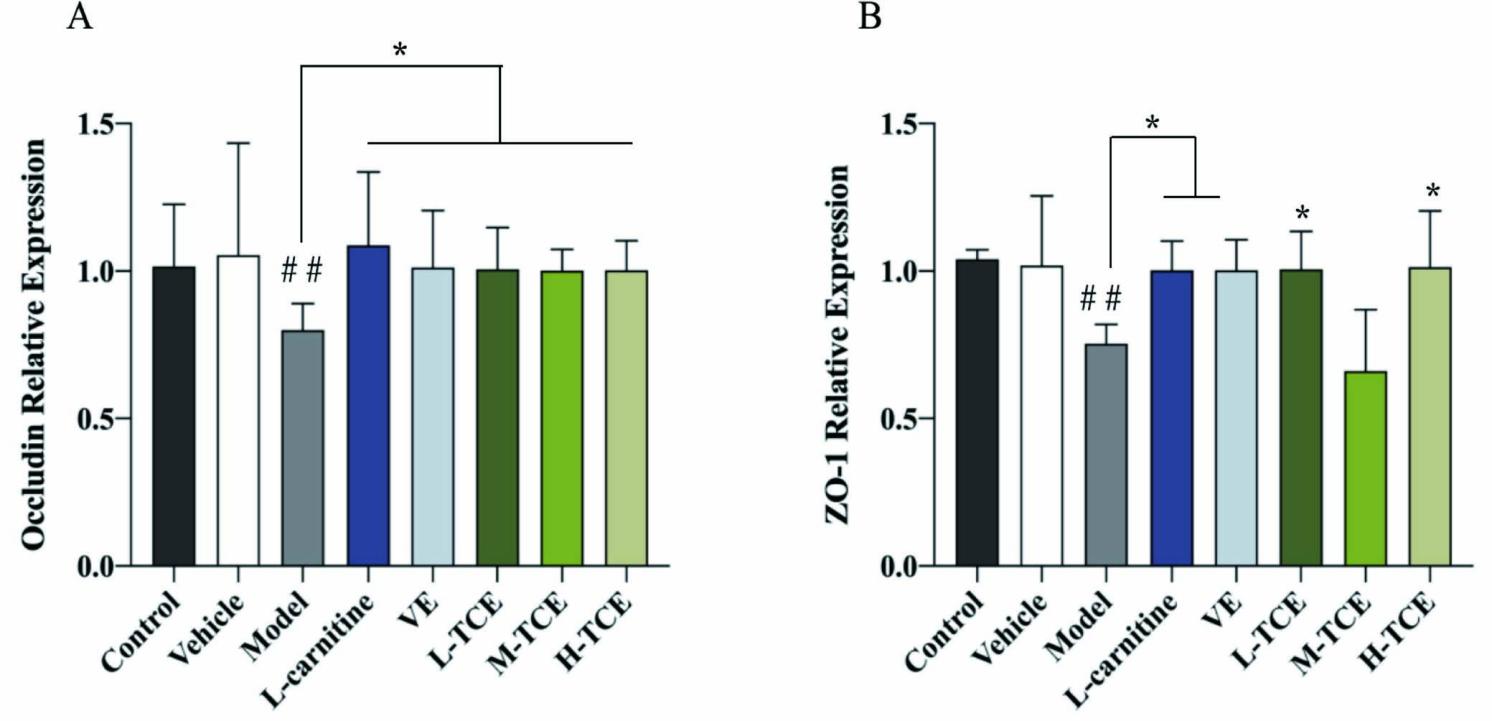


S3 The relative expression levels of 2 key BTB integrity-related genes. A. *Occludin*; B. *ZO-1*; Means ±SD; n = 6; **P* < 0.05, compared with the model group; ***P* < 0.01, compared with the model group; ^##^*P* < 0.05, compared with the control group.
